# Long-term single-cell imaging and simulations of microtubules reveal principles behind wall patterning during proto-xylem development

**DOI:** 10.1038/s41467-021-20894-1

**Published:** 2021-01-28

**Authors:** René Schneider, Kris van’t Klooster, Kelsey L. Picard, Jasper van der Gucht, Taku Demura, Marcel Janson, Arun Sampathkumar, Eva E. Deinum, Tijs Ketelaar, Staffan Persson

**Affiliations:** 1 School of Biosciences, University of Melbourne, Parkville, VIC 3010 Australia; 2 Max Planck Institute of Molecular Plant Physiology, Am Muehlenberg 1, 14476 Potsdam, Germany; 3 Laboratory of Cell Biology, Wageningen University, Wageningen, The Netherlands; 4 Physical Chemistry and Soft Matter, Wageningen University, Wageningen, The Netherlands; 5 School of Natural Sciences, University of Tasmania, Hobart, 7001 TAS Australia; 6 Graduate School of Biological Sciences, Nara Institute of Science and Technology, Ikoma, Nara 630–0192 Japan; 7 Mathematical and Statistical Methods (Biometris), Wageningen University, Wageningen, The Netherlands; 8 Department for Plant and Environmental Sciences, University of Copenhagen, 1871 Frederiksberg C, Denmark; 9 Copenhagen Plant Science Center, University of Copenhagen, 1871 Frederiksberg C, Denmark; 10 Joint International Research Laboratory of Metabolic and Developmental Sciences, State Key Laboratory of Hybrid Rice, School of Life Sciences and Biotechnology, Shanghai Jiao Tong University, Shanghai, China

**Keywords:** Plant sciences, Plant cell biology, Plant cytoskeleton

## Abstract

Plants are the tallest organisms on Earth; a feature sustained by solute-transporting xylem vessels in the plant vasculature. The xylem vessels are supported by strong cell walls that are assembled in intricate patterns. Cortical microtubules direct wall deposition and need to rapidly re-organize during xylem cell development. Here, we establish long-term live-cell imaging of single *Arabidopsis* cells undergoing proto-xylem *trans*-differentiation, resulting in spiral wall patterns, to understand microtubule re-organization. We find that the re-organization requires local microtubule de-stabilization in band-interspersing gaps. Using microtubule simulations, we recapitulate the process in silico and predict that spatio-temporal control of microtubule nucleation is critical for pattern formation, which we confirm in vivo. By combining simulations and live-cell imaging we further explain how the xylem wall-deficient and microtubule-severing KATANIN contributes to microtubule and wall patterning. Hence, by combining quantitative microscopy and modelling we devise a framework to understand how microtubule re-organization supports wall patterning.

## Abstract (web summary)

Plant cell wall formation is directed by cortical microtubules, which produce complex patterns needed to support xylem vessels. Here, the authors perform live-cell imaging and simulations of Arabidopsis cells during proto-xylem differentiation to show how local microtubule dynamics control pattern formation.

## Introduction

The plant vasculature contains xylem cells that are organized in interconnected tubular networks, to enable efficient water distribution to plant organs and to support plant stature^1^. All plant cells are surrounded by primary cell walls, which dictate growth direction. Xylem cells are reinforced by an additional wall layer, referred to as a secondary wall, which is deposited in local thickenings that form highly ordered spatial patterns^2^. Xylem cells subsequently undergo programmed cell death, which leads to the clearing of their cytoplasmic content and the resulting formation of a hollow tube that provides the water-conducting capacity of vascular plants^3^.

Besides the phenolic compound lignin, which renders secondary walls strong and waterproof, the major load-bearing component of plant cell walls is cellulose; a β-1,4-linked glucan. Cellulose is synthesized by cellulose synthase complexes (CSCs) that span the plasma membrane^4^. Nascent cellulose chains coalesce via hydrogen bonds, get entangled in the cell wall and further synthesis thus forces the CSCs to move in the membrane^5^. The CSC delivery to, and locomotion within, the plasma membrane is guided by cortical microtubules that presumably are associated with the plasma membrane^6–9^ and by cellulose microfibrils when CSCs temporarily lost microtubule guidance^10^. Cortical microtubules thus directionally and spatially template the cellulose synthesis machinery during cell wall deposition.

Microtubules undergo dynamic re-organization in response to environmental, developmental and physical cues^11–13^. Spatial control of microtubule arrays can be mediated by small GTPases termed *Rho of plants* (*ROP*s) in *Arabidopsis*, which are located in the cytoplasm and at the plasma membrane^14,15^. For instance, a *ROP11*-based reaction–diffusion mechanism drives localized secondary wall thickenings during differentiation of meta-xylem (MX) cells, which produce pitted wall patterns. This mechanism involves local activation of ROP11 at the plasma membrane and recruitment of microtubule depolymerizers, such as microtubule depletion domain (MIDD)1 and kinesin (KIN)13A^16–18^. The microtubule-associated proteins (MAPs) IQ-domain (IQD)13 and IQD14, and CORTICAL MICROTUBULE DISORDERING (CORD)1 control the shape of the active ROP11 domains, whereas BOUNDARY of ROP domain (BDR)1 and WALLIN (WAL) direct actin filaments to the border of the active ROP11 domain^19–21^.

In contrast to MX, the proto-xylem (PX) (cells that undergo differentiation when the surrounding tissue still elongates) consists of periodic or spiralling secondary wall bands. Mutations in the genes important for pit patterning in MX, i.e., ROP11, ROPGAP3/4, ROPGEF4/7, IQD13 and IQD14, CORD1, BDR1 and WAL, do not cause obvious PX wall defects, indicating a different regulation for periodic band patterning than for pit formation^16,19–21^. To support PX band patterning, microtubules undergo a transition from a diffuse array with variable microtubule orientations into a banded array where microtubule orientations are homogeneous^9,22,23^. From these studies, it is clear that cortical microtubules mimic the secondary wall patterns^2^ and template secondary wall cellulose deposition^9,22,23^. Nevertheless, the principles by which the dynamic microtubule network is re-organized during the transition from primary to secondary wall deposition in PX remain elusive. In addition, the transition period of microtubule array configurations during PX and MX formation is not well defined.

Xylem differentiation occurs in a sequential manner along the central axis of roots and shoots, which is buried underneath several cell layers. Detailed PX formation is therefore difficult to visualize. However, Yamaguchi et al.^24^ generated an elegant system in which PX formation can be induced in cells that typically do not make secondary walls in *Arabidopsis*^25^. Here, the PX inducing “master” regulator VASCULAR NAC-DOMAIN (VND)7 is under control of a chemically inducible glucocorticoid-receptor element and can drive ectopic PX differentiation upon induction. Recently, this system was used to investigate the behaviour of secondary wall cellulose synthesis, to measure the impact of CELLULOSE SYNTHASE INTERACTING 1 on this process and to assess coordination between transcript and metabolite changes, during PX formation, respectively^9,22,23,26,27^. This approach is thus of great aid in understanding the transition from primary to secondary wall formation during PX development.

Here we establish long-term, single live-cell imaging of *Arabidopsis* epidermal hypocotyl cells undergoing PX *trans*-differentiation. We quantitatively analyse microtubule dynamics parameters over time and use these parameters as input for computer simulations to understand the processes that drive the rearrangement of the microtubule array during PX formation.

## Results

### Microtubules re-organize rapidly during PX formation

**Fig. 1:**
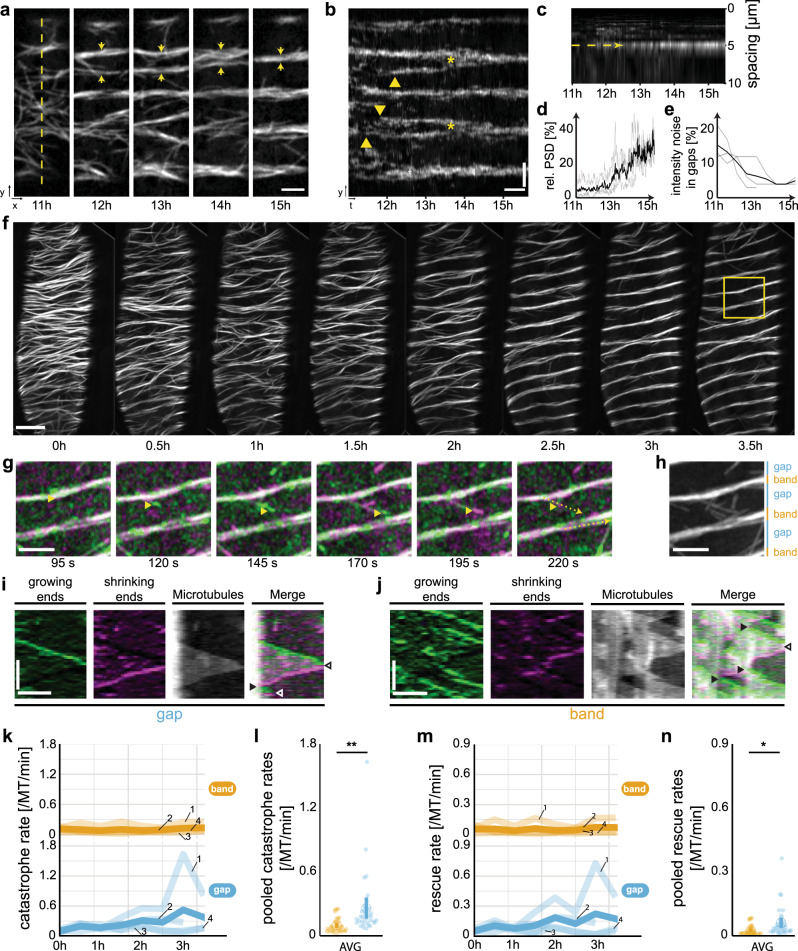
The inducible VND7 system drives ectopic microtubule rearrangements. **a** Long-term time-lapse recording of microtubule rearrangements at low-temporal resolution in an induced hypocotyl cell. Scale bar = 3 µm. Microtubule bands emerge close to each other but merge into one band over time (arrows). **b** Kymograph along dashed line in **a** reveals the emergence (arrowheads) and merging (asterisks) of microtubule bands. Scale bars = 30 min and 3 µm. **c** Periodogram of the kymograph in **a** reveals the development of a dominant microtubule spacing of 5.3 ± 0.5 µm (mean ± SEM, 6 cells, 4 seedlings). **d** The relative power spectral density (PSD) measured at the dominant spacing of four individual cells (grey lines) increases from 5.5 ± 0.3% (thick line, mean ± SEM) to 26.9 ± 0.5% within 2 h. **e** Microtubule noise (i.e., intensity s.d. normalized to sum intensity) decreases rapidly in gaps from 15 ± 5% (thick line, mean ± SD) to 5 ± 1%. **f** Long-term time-lapse recording at high temporal resolution of YFP-labelled microtubules in an induced hypocotyl cell. Scale bar = 5 µm. **g** Image series of box in **f** colour-coding for growing (green) and shrinking (magenta) microtubule ends. A dynamic microtubule end is highlighted by arrowheads. Scale bar = 5 µm. **h** Classification of microtubule bands (orange) and gaps (blue). **i**, **j** Kymographs along the dashed lines in **g**. In gaps, growing microtubule ends often undergo catastrophes (empty arrowheads) and rarely rescues (filled arrowheads). In contrast, microtubule ends in bands predominantly grow along the band axis and are often rescued after catastrophe. Scale bars = 2 µm and 100 s. **k**–**n** Quantification of microtubule dynamics in four time series’ (fine lines, numbered in **k** and **m**, thick line = time average) divided into bands (orange) and gaps (blue): **k**, **m** individual and **l**, **n** pooled catastrophe rates *r*_cat_ and rescue rates *r*_res_, respectively. Data pooled from 35 time points from four cells (AVG); means ± 95% confidence intervals. Statistics: Welch’s unpaired, two-sided *t*-test, *p* = 0.0021 (**l**) and *p* = 0.0107 (**n**).

To study the behaviour of the microtubule array upon induction of PX formation we used an mCHERRY-TUA5 microtubule reporter line^22^ in the VND7-inducible background (Supplementary Fig. 1a–d). We classified the *trans*-differentiation progression into early, mid, and late stages based on microtubule array changes^9^, using anisotropy measurements (Supplementary Fig. 1e)^28^. These measurements revealed an increased progression of array anisotropy during early, mid, and late stages of PX formation. However, this classification of array patterns is based on ‘snapshots’ of the microtubules and thus ignores their dynamic behaviour that drives the re-organization. To assess the microtubule dynamics, we developed an automated image acquisition script and optimized our live-cell imaging procedure by implementing a sample chamber that blocked water evaporation, which allowed us to acquire long-term recordings of single cells. The automated script enabled temporal control of the time lapses. We initially observed that microtubule bands emerged ~12 h after VND7 induction. To capture the complete array rearrangements, we therefore started detailed observations before changes to the microtubule array were visible (typically 6–11 h after induction). We first recorded single images of microtubules every 30 s for 5 h in a low-temporal-resolution experiment (Fig. 1a and Supplementary Movie 1). We found that several microtubule bands and spirals formed simultaneously over the entire cell cortex, including the backside of the cells (Supplementary Fig. 1f). This initial pattern was occasionally readjusted during the following 2 h (Fig. 1a, yellow arrows). Readjustments involved gradual shifting of microtubule bands (Fig. 1b, arrowheads) that sometimes led to two neighbouring bands merging (Fig. 1b, asterisks). After such readjustments, equally spaced and parallel bands remained at fixed positions throughout the rest of the time course.

To gain insight into the periodicity maintenance during microtubule array patterning, we performed periodogram analysis of the intensity profile along the cell’s growth axis (dashed line in Fig. 1a). A periodogram provides an estimate for how a particular (spatial) frequency contributes to a fluctuating signal. We calculated the power spectral density (PSD) of the intensity profile in Fig. 1a and plotted it against the spatial frequencies in the profile (Fig. 1c). We found that the PSD initially was evenly distributed, indicating contributions from all spatial frequencies to the intensity profile as expected from a dispersed array. However, the PSD subsequently peaked at spatial frequencies corresponding to ~5 µm (dashed arrow in Fig. 1c). By plotting the temporal development of the peak in the PSD, we estimated that the rearrangement of the microtubule array, i.e. from a diffuse array into periodic bands, occurred within one to 2 h (Fig. 1d). The microtubule intensity gradually decreased in the gaps between the bands, indicating continuous microtubule removal from the gaps (Fig. 1e). Hence, although the VND7-driven *trans*-differentiation takes relatively long (up to several days; Supplementary Fig. 2)^24,29^, the microtubule array re-organization occurs rapidly.

### Microtubule dynamics differ between bands and gaps

The low-temporal resolution recordings enabled us to observe global microtubule re-organization during PX formation. However, individual microtubules undergo dynamics that occur on the timescale of a few seconds and are thus not captured in the long-term recordings. We therefore increased the temporal resolution to 5 s intervals, taken for 5 min every 30 min. To avoid photobleaching, we generated a YFP-TUA5 expressing VND7-inducible line and used it instead of mCHERRY-TUA5. Similar to above, we found that the total microtubule intensity differed between bands and gaps during the time course (Supplementary Fig. 3a). Whereas the intensity remained relatively constant in bands (94 ± 24% of initial band intensity counts; mean ± SD measured throughout whole time course), it decreased within an hour after the start of the recording (48 ± 13% of initial gap intensity counts) in the gaps and remained low afterwards (37 ± 15%). The intensity ratio between bands and gaps, which can be interpreted as a measure for band progression, consequently increased throughout the time course.

We were able to record four long-term image series that captured the complete *trans*-differentiation process (Fig. 1f, Supplementary Figs. 4–7 and Supplementary Movies 2–5). We found that microtubules underwent frequent depolymerization due to catastrophes when they were located in gaps (Fig. 1g–i). By contrast, the majority of microtubules in the bands appeared to grow, with low depolymerization frequency (Fig. 1j). To quantify microtubule dynamics in bands and gaps, we used image processing to detect growing and shrinking microtubule ends in the time-lapse recordings (Supplementary Fig. 8)^30^. This approach allowed straightforward quantification of microtubule growth (*v*_+_) and shrinkage (*v*_−_) speeds, and catastrophe (*r*_cat_) and rescue (*r*_res_,) rates, which are the most relevant parameters to characterize microtubule dynamics^31,32^. We divided the cortical area into regions that developed into bands and gaps (Fig. 1h) and determined the microtubule dynamic instability parameters (Supplementary Table 1). In bands, microtubules grew and shrank on average with a speed of 53 ± 15 nm/min and −80 ± 19 nm/min, respectively, whereas in gaps, microtubules grew and shrank on average with a speed of 54 ± 15 nm/min and −86 ± 20 nm/min (means ± SD, 35 time points from four time series). Neither microtubule growth nor shrinkage speeds were significantly different between bands and gaps throughout the microtubule re-organization process (Supplementary Fig. 3b). We further measured *r*_cat_ and *r*_res_ and found both to be significantly larger in gaps as compared to bands (Fig. 1k–n). Microtubules in the band regions underwent catastrophes and rescues at average rates of 0.096 ± 0.066 and 0.054 ± 0.048 events per microtubule end per minute, respectively. Significantly higher rates and variations were found in gaps with average *r*_cat_ and *r*_res_ of 0.258 ± 0.282 and 0.120 ± 0.138 events per microtubule end per minute, respectively (*p*_band_ < 0.002 and *p*_gap_ < 0.011; Welch’s unpaired, two-sided *t*-test). Although we observed significant changes in microtubule density and catastrophe/rescue rates in bands vs. gaps across the four time series, we also observed variations in microtubule re-organization speed and dynamics between the individual cells (Supplementary Figs. 4–7). Taken together, these results indicate that microtubules display more vigorous dynamics in the gaps as compared to bands.

### A conceptual framework to simulate microtubule band formation

To gain insight into how microtubule dynamics contribute to band formation, we proceeded to establish a framework that could simulate these patterns. To do this, we drew inspiration from three theory-derived concepts^33^: (i) the so-called control parameter *G*, which collapses the microtubule dynamic instability parameters and nucleation rate into a single number, (ii) the average microtubule length in absence of interactions *L*_0_, and (iii) the average microtubule lifetime *τ* (Methods and Supplementary Note 1). The control parameter *G* is a theory-derived quantity that combines the microtubule dynamic instability parameters *v*_+_, *v*_–_, *v*_t_ (the minus-end retraction velocity), *r*_res_, *r*_cat_ and *r*_n_ (the nucleation rate) into a single number (Eq. (1) in the Supplementary Note 1). *G* can be interpreted as the negative ratio of two length scales: the ‘interaction distance’ or average distance between consecutive encounters of growing microtubule tips and the ‘average microtubule length’ in the absence of microtubule–microtubule interactions. The closer *G* approaches zero, the more interactions will take place during an average microtubule lifetime and, hence, the larger the propensity of the array to align spontaneously^31,33,34^. Using the measured microtubule dynamic instability parameters obtained from our time series recordings and an assumed isotropic nucleation rate, we found that *G, L*_0_ and *τ* were approximately equal between our defined band and gap regions before induction, as expected for a diffuse microtubule array (Supplementary Figs. 4–7 and 9). However, differences became evident upon microtubule band formation. We estimated *τ* to be 28.8 ± 16.7 min in bands (*τ*_band_; means ± SD, *n* = 35 time points), whereas in the gap *τ* was significantly shorter: 11.0 ± 5.3 min (*p* < 0.0001, Welch’s unpaired, two-sided *t*-test, Supplementary Fig. 9c). We used these values to calculate ratios between gaps and bands, *G*_gap_/*G*_band_ and *τ*_band_*/τ*_gap_, to compare the course of the re-organization process in different cells. We found that each cell displayed a phase of peaking ratios but that these peaks occurred at different time points (Supplementary Figs. 4–7 and 9d, e). The ratios of *G*- and *τ*-values followed very similar curves, reflecting the near inverse relationship between the definitions of *G* and *τ* (Supplementary Note 1). Whereas the progression of array behaviour differed between cells, all ratios started close to 1, indicating a diffuse microtubule array, and maintained similar overall trends.

### Microtubule nucleation controls band patterning in silico

To investigate whether the microtubule-based parameters (*G*, *τ* and *L*_0_) are sufficient to separate a diffuse microtubule array into bands and gaps, and thus to mimic PX *trans*-differentiation, we performed computer simulations (Supplementary Note 1). To this end, we used an extended version of the ‘*Cortical Sim*’ software^31^ and a parameter set based on the measurements outlined above (Supplementary Table 1). Cortical Sim renders dynamic microtubules as growing and shrinking line segments on a closed surface representing the cell cortex. These in silico ‘microtubules’ interact through frequent encounters, occurring as growing microtubules impinge on obstructing ones. The outcome of such collisions depend on the relative angle: ‘*zippering*’ or ‘*bundling*’ (continued growth along the obstructing microtubule) for angles <40° and ‘*crossover*’ (continued growth without change of direction) or ‘*induced catastrophes*’ (switch to shrinkage) for larger angles^35^.

**Fig. 2:**
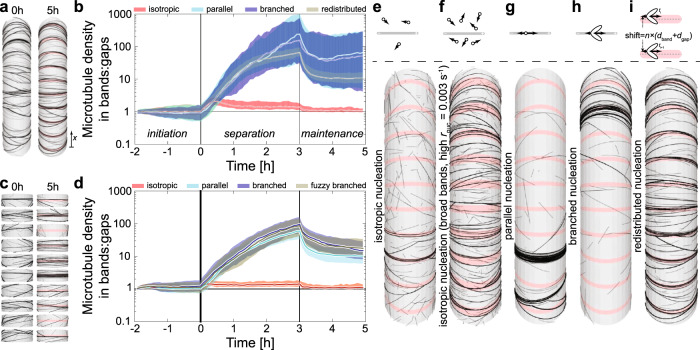
Simulation of dynamic microtubules reproduces patterning into bands and gaps. **a** Design of ten-banded cells: microtubules (black lines) are simulated to undergo dynamics on the surface of cylindrical cells. Ten bands (pink) separated by gaps were distributed evenly over the cell surface. The separation phase runs from *T* = 0 h (left) to 3 h, followed by a 2 h maintenance phase (*T* = 5 h, right). **b** Degree of separation for four different nucleation modes. Lines and margins represent medians ± 16% and 84% percentiles from more than 100 individual simulation runs. **c** Improved design using ten, independent ‘single-band’ models: single bands including their gap environment with a total length of 6 µm at *T* = 0 h (left) and *T* = 5 h (right). All other parameters remained as in **a**. **d** Degree of separation for ‘reconstituted’ structures for four different nucleation modes. Lines and margins represent medians and 16% and 84% percentiles, respectively, for more than 100 ‘reconstituted’ structures comprised ten unique single-band models obtained from 2000 independent simulations. **e**–**i** Top row: schematic representations of the five different nucleation modes: isotropic (**e**), isotropic with threefold higher nucleation rate and 2 µm-wide bands (**f**), parallel (**g**), branched (**h**) and redistributed nucleation (**i**). A microtubule is depicted as a grey rod with plus and minus ends. Arrows indicate the direction of polymerization from a nucleation site. Elliptic areas depict orientation bias for branched nucleation of ~35° relative to the microtubule axis. Redistributed nucleation involves determination of nucleation site as with parallel nucleation, followed by a shift of a random integer times the length of the repeating unit (*d*_band_ + *d*_gap_) leading to global redistribution of the nucleation sites, while maintaining relative position to the nearest band centre. Lower row of panels shows snapshots of representative simulations for each nucleation mode.

For our simulations, we used 60 µm-long and 15 µm-wide cylindrical cells (Fig. 2a). Along the long axis, we subdivided the cell into 5 µm-wide gaps, consistent with our PSD measurements (Fig. 1c), and 1 µm-wide bands (pink region in Fig. 2a, right), to which we applied different parameters based on the experimentally observed microtubule dynamic instability parameters in the respective regions (Supplementary Table 1). To create a transverse and diffuse microtubule array, we implemented a 2 h initiation phase where the microtubule dynamic instability parameters were identical in bands and gaps. Because of the variation among the quantified cells, the resultant computer simulations produced highly different outcomes (Supplementary Figs. 4–7 and 9f). We therefore decided to use a simplified approach in which we attempted to generally describe the band formation process and determine key properties that lead to robust separation. We thus created a ‘caricature’ dataset, which we based on the typical values obtained from our experiments (Supplementary Table 1, bottom). We then attempted to simulate the band formation by implementing two temporal phases: (i) separation, where a large difference of the microtubule dynamic instability parameters, i.e., a large difference in *G*, between bands and gaps is applied and (ii) maintenance, where a much smaller difference is applied. Although band-associated *G*-values increased in three of four cells (Supplementary Fig. 9b), these values fluctuated. As a worst-case scenario, we kept them constant throughout the simulations, at a level representative of ‘early’, i.e., prior to band separation, microtubule arrays. From this baseline, we used the catastrophe rate *r*_cat_ in the gaps to tune the gap-associated *G*-value and hence the overall ratio in *G*_gap_/*G*_band_. We did this by increasing *r*_cat, band_ by an integer factor (default: 5× during separation and 2× during maintenance phase). The resulting ratios of *G* were similar to the large ratios observed in our experimental data (Supplementary Table 1). As a key measure, we monitored the ‘*degree of separation*’ (= ratio of microtubule densities in bands vs. gaps) over time. To assess the impact of different parameter settings, we first simulated them assuming isotropic microtubule nucleation, i.e., with randomly distributed locations and orientations. This yielded only a very low degree of separation (Fig. 2b, e), regardless of nucleation rate (Fig. 2f: threefold increased). For more natural behaviour, we then used that microtubule nucleation occurs predominantly from existing microtubules in diffuse array configurations^36^. This assumption resulted in a strongly increased degree of separation (Fig. 2b, g–h). Here, the relative nucleation angles—parallel (default; new orientation roughly [anti-]parallel to parent microtubule) or branched (new orientation exactly [anti-]parallel to parent or (40%) with a mean relative angle of 35°)—had little impact.

The strong effect of microtubule-bound nucleation on the degree of separation can be understood as a positive feedback loop: a local increase in microtubule density attracts more nucleations, resulting in a further increase of the local microtubule density^37^, enhancing separation ~10- to 20-fold (Supplementary Fig. 10 and Supplementary Note 1). In our simulations, the microtubule density-based competition for nucleations is global, i.e., over the whole cell length, which typically resulted in the over-amplification of a single or a few dominant bands (Fig. 2g, h and Supplementary Fig. 11), comparable to an unsolved issue in simulations of homogeneous arrays^37,38^. This unrealistic behaviour indicated that in planta, the competition for nucleation complexes must be local rather than global. To demonstrate the importance of local competition, we artificially redistributed the nucleation sites randomly retaining their distance to the centre of the nearest band (Fig. 2i). As microtubules do not jump from one location to another in the cell cortex, we decided to separate the cell into short, single-band pieces that would offer a more comprehensible way of bypassing the global competition issue. To compare such ‘single-band’ models to the previously simulated ten-banded cells, we combined ten independent single-band models to form a ‘reconstituted’ ten-banded structure (Fig. 2c and Supplementary Note 1), which yielded similar statistics (Fig. 2b, d). These results suggest that band formation is restricted to a regime where the nucleation of microtubules preferentially occurs from the emerging bands and that nucleation complexes are locally recruited to the bands. Consistent with this, we found that microtubules rapidly re-populated forming bands after cold-induced depolymerization (Supplementary Fig. 12). In addition, the model allowed us to explore effects of modifying the *G* and *τ* parameters (Supplementary Note 1 and Supplementary Figs. 13 and 14).

### Microtubule nucleation is locally recruited to bands in planta

**Fig. 3:**
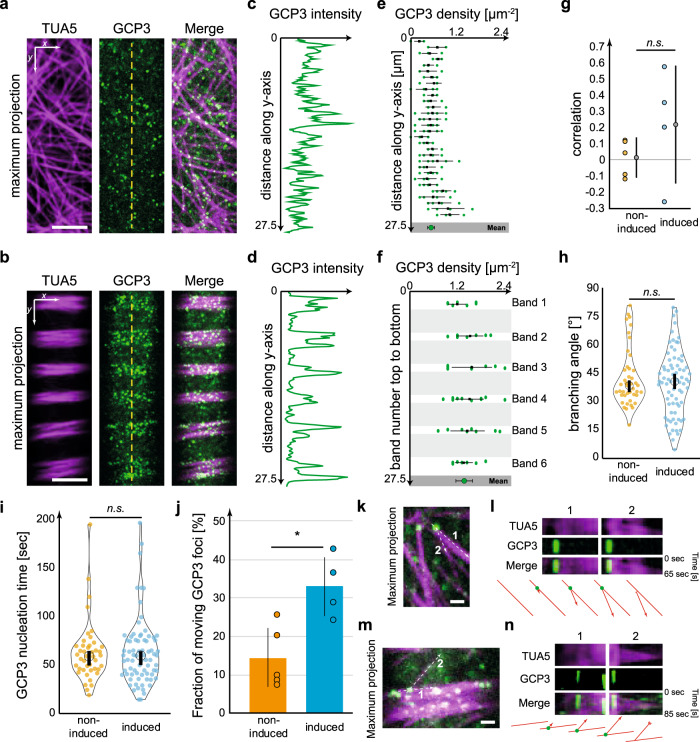
GCP3 nucleation complexes distribute evenly across developing bands during proto-xylem formation. **a**, **b** Maximum projections of dual-labelled mCH-TUA5 GCP3-GFP in non-induced cells (**a**) and during PX formation (**b**). Scale bar = 5 µm. **c**, **d** Intensity along the lines in **a** and **b**. **e**, **f** Cortical GCP3 density along the *y*-axis of non-induced cells (**e**) and in microtubule bands of induced cells (**f**). Each dot refers to a density measurement, black squares and lines represent means ± SD for each section of cortex. Pooled measurements (bottom) average to a GCP3 density of 0.56 ± 0.19 foci per µm^2^ (mean ± SD, 155 measurements, 5 cells) in non-induced cells (**e**) and to 1.41 ± 0.43 foci per µm^2^ (56, 4, *p* < 0.0001, Welch’s unpaired, two-sided *t*-test) in induced cells. **g** GCP3 intensity is uncorrelated along the *y*-axis indicating an even distribution of GCP3 foci across bands (mean ± 95% confidence intervals; *p* > 0.91, Welch’s unpaired, two-sided *t*-test). **h**, **i** Branching angle and nucleation time of microtubules from GCP3 foci located on parent microtubules are identical in non-induced (orange) and induced cells (blue), respectively (means ± 95% confidence intervals, *p* > 0.37 and *p* > 0.97, for 43 and 76 new microtubules and GCP3s foci, from 5 and 4 cells, respectively). **j** Proportion of moving GCP3s in non-induced (orange) and induced (blue) cells (mean ± SD, 43 and 76 GCP3s from 5 non-induced and 4 induced cells, *p = 0.0142, Welch’s unpaired, two-sided *t*-test). **k**–**n** Maximum projections showing microtubules nucleated from parent microtubules in non-induced cells (**k**) and induced cells (**m**). Dashed lines indicate parent (1) and new (2) microtubules used to generate kymographs (**l**, **n**). Scale bar = 1 µm. Panels **l** and **n** show kymographs of stationary GCP3s during nucleation of new microtubules for non-induced (**l**) and induced (**n**) cells. Below: schematic representation of the nucleations shown in the kymographs. red: microtubules, green: GCP3.

To investigate how our simulation predictions of nucleation distribution compared with microtubule nucleation in planta, we used plants expressing the green fluorescent protein (GFP)-labelled γ-tubulin complex protein (GCP)3^39^. As microtubule nucleation is challenging to visualize in the tissue that naturally undergoes xylogenesis, we instead crossed the GCP3 plants into the mCHERRY-TUA5 VND7 background. We found that GCP3 foci coincided with cortical microtubules in agreement with Nakamura et al.^39^, leading to an even distribution of GCP3 foci in non-induced cells and in the bands of induced cells (Fig. 3a–d). We measured the density of GCP3 foci and found that induced cells had significantly higher foci density in bands as compared to gaps (Fig. 3d), and compared to the even distribution observed at the cortex of non-induced cells (Fig. 3e, f). We did not find any statistical differences in the GCP3 density between neighbouring bands (Fig. 3f). The GCP3 intensity gradient along the long axis of induced and non-induced cells was, furthermore, not significantly different from zero (Fig. 3g). These data are thus in good agreement with our simulation results.

We next investigated whether the GCP3s promote microtubule nucleation during *trans*-differentiation. We focused on microtubules at the edge of bands, as nucleation events here were readily observable as compared to microtubules located in the centre of bands and compared those with microtubule nucleation in non-induced cells. We found that new microtubules were branched from parent microtubules at angles of ~40° in both induced and non-induced cells (Fig. 3h). Similarly, microtubule nucleation typically occurred from GCP3 foci that had dwelled on microtubules for on average 60 s (Fig. 3i). In non-induced cells, the vast majority of nucleating GCP3s were stationary (86 ± 8% of all foci moved <100 nm within 25 s, mean ± SD, 5 cells, Fig. 3j, k, l), whereas this was the case in only 67 ± 8% of the cases in induced cells (Fig. 3j, m, n). Interestingly, in induced cells the remaining GCP3s displayed linear movements along microtubules in the bands (Supplementary Movies 7–9). These movements resulted in larger variability of microtubule branching angles as compared to that in non-induced cells (Fig. 3h). Closer inspection revealed that the linear GCP3 movements were due to anti-parallel encounters of polymerizing, newly nucleated microtubules, followed by microtubule–microtubule contraction, i.e., microtubules sliding along each other, with velocities of 14 ± 8 nm/s (mean ± SD, 35 GCP3s from 4 induced cells). This phenomenon was not observed in non-induced cells (Supplementary Movie 6). Together, these results indicate that our assumptions of microtubule nucleation in the simulations agree with how nucleation occurs during microtubule separation. Furthermore, anti-parallel microtubule–microtubule contractions seem to be an overlooked process that might assist in microtubule organization in bands.

### Microtubule band formation is impaired in the *ktn1-2* mutant

**Fig. 4:**
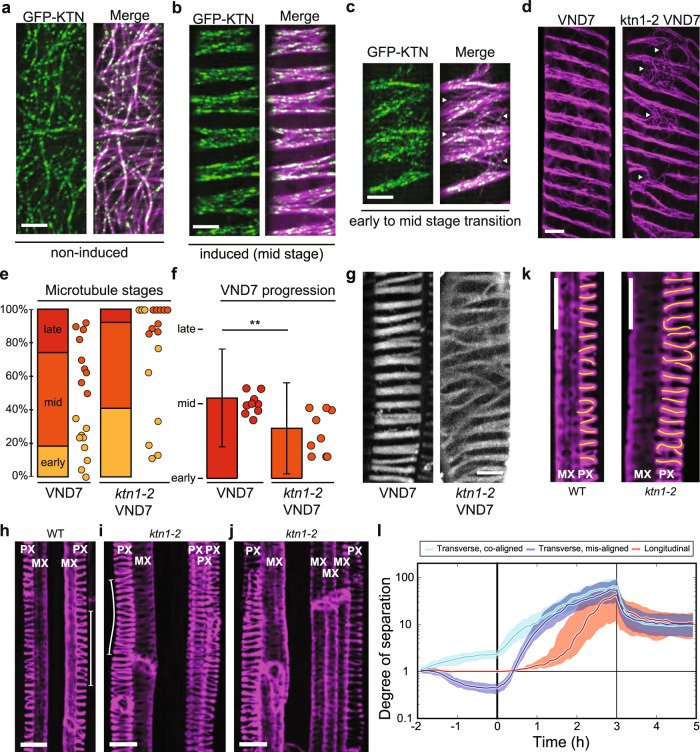
Fidelity and speed of microtubule band formation depends on KATANIN. **a**–**c** Maximum projection of dual-labelled mCh-TUA5 and GFP-KTN seedlings in non-induced cells (**b**) and during PX formation (**b**, **c**). KTN1-GFP localizes majorly to forming microtubule bands during early (**b**) and mid (**b**) stages of PX formation. Scale bars = 10 µm. **d** Maximum projections of mCh-TUA5 VND7 during PX formation in wild-type (left) and in *ktn1-2* (right). Arrowheads in **c** and **d** point toward regions with incomplete band separation. Scale bar = 5 µm. **e** Fraction of cells classified into early, mid and late stages of PX formation for induced mCh-TUA5 VND7 (left) and *ktn1-2* VND7 (right). Dots depict fractions of early and mid-stages of nine seedlings for each genotype. **f** Relative progression of VND7 (left) and *ktn1-2* VND7 (right). Microtubule arrays in VND7 are significantly more progressed (2.1 ± 0.7, mean ± SD, 174 cells from 9 seedlings) compared to the *ktn1-2* VND7 (1.7 ± 0.6, 127, 9, **p = 0.0042, Welch’s unpaired, two-sided *t*-test). Dots depict VND7 progression for nine seedlings for each genotype. **g** Maximum projection of stained secondary walls of induced wild type (left) and *ktn1-2* (right) 48 h after induction. Scale bar = 10 µm. **h**–**k** Basic Fuchsin-stained wild-type (h) and *ktn1-2* (i-j) xylem files in 6-day-old roots. In *ktn1-2*, an altered arrangement of PX (**i**) and MX (**j**) vessels is observed, PX vessel thicknesses vary (compare vertical lines in **h** and **i**) and cell wall bands are wider and more disordered (**k**, right) as compared to wild type (**k**, left). Band shapes are indicated by yellow lines. Scale bars = 10 µm. **l** Band separation is fastest for initially co-aligned transverse arrays (cyan and blue) and slowest for initially longitudinal arrays (red). Simulations assuming default parallel nucleation and no KTN activity (*r*_x_ = 0 s^−1^). Lines and margins represent medians ± 16% and 84% percentiles.

We next investigated how defects in microtubule dynamics and organization influenced PX band separation. We therefore focused on the microtubule-severing protein complex KATANIN (KTN), which is important for secondary wall production and microtubule alignment^40,41^. The KTN complex is a hexamer of KTN1-KTN80 heterodimers^42^. The single copy of KTN1 is responsible for microtubule-severing whereas four KTN80 isoforms confer targeting to microtubule crossovers. Consistent with a role of KTN in PX formation, KTN1 and at least one isoform of KTN80 were expressed and upregulated ~8 h after VND7 induction (Supplementary Fig. 15a)^26^, which corresponds well with the time point when microtubules re-organize prior to band formation. To corroborate these data, we crossed a functional KTN1-GFP^12^, driven by its native promoter, into our mCHERRY-TUA5 marker line in the VND7 background. We found that in non-induced cells, KTN1-GFP co-localized dynamically with microtubules (Fig. 4a). In induced cells, we found that KTN1-GFP strongly labelled the forming microtubule bands during the mid-stage of PX formation (Fig. 4b, c). These data confirm that KTN is associated with microtubule bands during PX formation and may be active during this process.

To obtain insight into how KTN affects PX microtubule band formation, we crossed a *ktn1-2* knockout mutant into our mCHERRY-TUA5 marker line in the VND7 background^12,39^. In agreement with previous work, we found that microtubules were more isotropically organized in the *ktn1-2* mutant (Supplementary Fig. 15b–d). Nevertheless, *ktn1-2* mutants were clearly able to undergo microtubule separation upon VND7 induction (Fig. 4d). However, the resulting bands displayed defects in alignment and were less coherent than in the wild type. We observed that some microtubule bands did not separate well from each other and maintained bridging microtubule clusters (arrowheads in Fig. 4c, d). When counting the number of cells in early, mid and late stages of PX formation (at 16–18 h after induction), we found that the *ktn1-2* cells were substantially delayed (Fig. 4e, f). Although the wild-type VND7 seedlings were on average between mid and late stages, the *ktn1-2* seedlings were not yet at mid-stage. When staining the secondary cell walls of wild-type and *ktn1-2* seedling cells in the VND7 background we found similar misarrangements of cell wall bands in *ktn1-2* (Fig. 4g) that closely matched the aberrations observed for the microtubule array. To confirm a functional role of KTN in ‘native’ xylem development, we stained the secondary walls of cleared wild-type and *ktn1-2* roots (Fig. 4h–k). We found aberrations in the xylem architecture of *ktn1-2* mutants, including differences in the number of PX (Fig. 4i) and MX (Fig. 4j) vessels, larger variations in vessel thickness (Fig. 4i, j), and wider and more disorganized cell wall bands in *ktn1-2* as compared to wild type (Fig. 4k). Taken together, these results indicate that KTN is not essential to drive microtubule band separation but does affect the fidelity and speed of this process, both in native and induced PX.

### Array orientation and anisotropy impact band formation in silico

To try to understand the KTN-induced changes in the band separation process, we included KTN-based microtubule severing at crossovers in our simulations^43^. We found that this KTN activity had little impact on the separation process, regardless of the type of nucleation mode (parallel, branched or ‘fuzzy branched’; Supplementary Fig. 16 and Supplementary Note 1). We next hypothesized that the main cause of delayed band formation in the *ktn1-2* mutant could be the impaired formation of well-aligned, anisotropic arrays prior and during the band separation. To investigate this, we altered the initiation phase to generate a wider range of initial array anisotropies and orientation angles at the start of the separation phase. To this end, we employed isotropic nucleation throughout the initiation phase. We also used two different values of the parameter *P*_cat_, the induced catastrophe probability for large angle collisions, during the initiation phase: *P*_cat_ = 0.5 (default), which yields mostly well-aligned arrays (Supplementary Fig. 17a–d), and tenfold reduced *P*_cat_ (0.05), which predominantly yields poorly aligned arrays (Supplementary Fig. 17e–h). We then computed the correlation coefficients between the separation rate (= relative increase of the degree of separation from *T* = 0 h) and (i) the degree of separation at *T* = 0 h, (ii) the degree of alignment (*S*_2_; a measure of anisotropy)^43^ at *T* = 0 h, and (iii) overall microtubule array orientation (*Θ*) at *T* = 0 h at multiple time points (Supplementary Fig. 17i). We found that for both data sets, initial array orientation correlated stronger with separation rate than anisotropy. The effect of orientation was, moreover, stronger for well-aligned arrays. During the first 40–50 min, there also was a strong correlation of separation rate with the initial degree of separation. To make a stronger case about the importance of the initial state, we then compared the behaviour of three specific subpopulations of the simulated cells: (i) longitudinal microtubules at *T* = 0 h, (ii) transverse microtubules at *T* = 0 h, with the largest microtubule density in the band region, and (iii) transverse microtubules at *T* = 0 h, with the largest microtubule density in the gaps (i.e., >1 µm away from the band). In all cases (with and without KTN and with three different nucleation modes), separation was established first in the already co-aligned transverse cells, then (after ~1 h) in the misaligned transverse cells and last in the longitudinal cells (Fig. 4l and Supplementary Fig. 18). These findings suggest that separation proceeds fastest if the initial state of the array matches the banding pattern well, i.e., is transversely aligned, and chance fluctuations in microtubule density match the bands. These results provide explanations why the *ktn1-2* mutant—which shows lower anisotropy and more disperse microtubule orientations across the cell cortex —displayed slower microtubule separation rates than the wild-type array. The observation that the microtubule bands in the *ktn1-2* mutant remained connected for longer periods also suggests that the KTN function plays a role in defining band-gap boundaries.

## Discussion

Microtubule patterns form the template for the deposition of cell wall components, which are typified during xylem differentiation. Our study shows that the behaviour of microtubules changes spatially and temporally during PX formation, and that local rather than global feedback for nucleation complexes is required for efficient separation of microtubules into bands and gaps. Furthermore, we identified that KTN is necessary for the rapid formation of finely ordered band patterns, a function probably based on the KTN-dependent formation of transverse microtubule arrays before the onset of the secondary wall-associated microtubule rearrangements.

There are clear similarities in PX and MX formation as cortical regions with a reduced microtubule density show absence of wall thickenings. In addition, we observed unchanged *v*_+_ and *v*_−_, but a temporal increase in *r*_cat_, in microtubule gaps, similar to that in microtubules of developing pits^44^. This similarity might indicate that microtubule removal from gaps and pits is driven by analogous mechanisms, perhaps via the recruitment of the microtubule depolymerizers *MIDD1* and *KIN13A*^16–18^, which are necessary for pit formation in MX. In fact, both genes are upregulated upon VND7 induction^25^. Contrary to the reduced microtubule rescue rates observed at pits^44^, we found an increase in the microtubule rescue rates in the band and gaps. In our simulation approach, we investigated the observed parameter differences with help of theoretical quantities computed from dynamic parameters^33^: *G*-values, related to microtubule length and stability, and average microtubule lifetimes (*τ*). We found that not only a sufficient difference between bands and gaps (peaks in the ratios *G*_gap_/*G*_band_ and *τ*_band_/ *τ*_gap_), but also sufficiently low lifetimes in the gap regions were required for timely separation (Supplementary Note 1). As long as these conditions were met, our results were robust under various parameter changes. Our simulations also indicated a need for increased nucleation in the bands, roughly evenly distributed among bands. These predictions were confirmed by the increased GCP3 density that we observed in all bands.

We observed that bands formed simultaneously across the *trans*-differentiating cells, indicating that inter-band spacing and orientation is dictated by a cell-wide pattern that has formed before, or during the onset of microtubule rearrangements. The existence of such a prepattern in PX has not been shown. Alternative explanations for microtubule band formation (that do not require a prepattern) propose MAPs, such as MAP65-1^45^, MAP70s^46^ and other MAPs^47^, to be the driver of microtubule bundling in developing xylem cells. Although evidence that such MAPs support microtubule banding is abundant, it remains unclear how MAPs support the uniform spacing between bands. It is thus tempting to speculate that this behaviour is instead driven by a Turing-like reaction–diffusion mechanisms that simultaneously patterns plasma membrane-associated factors—e.g., ROPs—into bands and spirals in silico^48^. This process, in conjunction with the ability to merge incoherent bands, occurred rapidly and appears crucial for accurate cell wall patterning. A computational study on ROP patterning suggested that microtubule arrays, by forming a transverse barrier to ROP diffusion, can only control ROP pattern orientation before straight bands appear. This indicates that microtubules need to be oriented transversely prior to the onset of band separation^48^.

In MX, microtubules confine the gap regions by acting as diffusion barriers for active ROP11^16,19^. Proteins that influence the properties of microtubule arrays might thus play an important role in fine-tuning the shape and fidelity of gaps in PX. To exemplify this mechanism, we focused on KTN and showed that it exerts a function during PX formation. Although *ktn1-2* mutants produced microtubule bands, their shape, relative positioning and timely appearance were altered. In our simulations, we started with highly aligned arrays that had a similar orientation as the predefined band regions, with thus few crossovers left for KTN severing. This starting point may not be reached in *ktn1-2* mutants, which generally has less ordered arrays^40,41^. We clearly observed the importance of the array state prior to band formation in our simulations with more variable initial arrays. There, the congruence between initial array and band pattern (as measured by various quantities) strongly affected separation rate and final state. These findings suggest that KTN impacts the patterning process through the array state prior to band formation^48^. If the *trans*-differentiation process contains a check point for a sufficiently aligned array with correct orientation, this may lead to additional delays in the *ktn1-2* mutant, which shows impaired ability to reorient to external cues^12,13^.

In addition, KTN1 functions in releasing microtubules from nucleating GCPs^39^ and in severing microtubule crossovers^49^. Without this function, microtubules that grow into gap regions at nucleation angles of ~40° may become more frequent. The resulting microtubules might occasionally bridge the gap regions, and further recruit other microtubules and stabilizing proteins, which may result in the formation of inter-band bridges. This may provide an explanation for the band-to-band microtubule crossovers in the *ktn1-2* mutant. The maintenance of microtubule bands is also driven by the ability to merge bands that are incoherent to the periodic band patterns. MAP70-5 may be a factor involved in delimiting microtubule bands, since it localizes to the periphery of microtubule bands^46^.

In summary, our study provides substantial new insight into how microtubules are re-arranged to sustain cell wall patterning. The simulation approaches may be extended to further predict microtubule-related functions to spatio-temporally control microtubule band separation and could also allow for similar assessments during other types of wall patterning.

## Methods

### Plant material and growth conditions

*Arabidopsis* (*Arabidopsis thaliana*, Columbia Col-0 ecotype) expressing 35S promoter-driven VND7-VP16-GR (VND7) and the 35S promoter-driven empty vector (EV) control line VP16-GR^24,25^ were crossed with the 35S promoter-driven YFP-TUA5^12^ and mCHERRY-TUA5 line^8^, respectively. We additionally crossed the mCHERRY-TUA5 VND7 line into the pKTN1::GFP-KTN1 in the *ktn1-2* mutant background^12^ to obtain the dual-labelled mCHERRY-TUA5 GFP-KTN1 VND7 line and the mCHERRY-TUA5 VND7 line in the *ktn1-2* mutant background. We also crossed the mCHERRY-TUA5 VND7 line into the pGCP3::GCP3-GFP line^39^ to obtain the dual-labelled mCHERRY-TUA5 GCP3-GFP VND7 line. The segregating F2 progeny was used for all experiments using the inducible dual-labelled marker lines whereas the F3 progeny was used for the homozygous *ktn1-2* mutant lines. Seeds were surface-sterilized by application of 1.25% bleach (Sodium hypo-chloride) and 0.1% Triton100 for 10 min followed by thorough washing with sterile water, then stratified for 2 days at 4 °C in the dark. Seeds were grown on ½ Murashige and Skoog medium (MS, Sigma-Aldrich, 0.8% micro-agar) supplemented with 1% (w/v) sucrose at pH 5.7. The plates were placed vertically in a 16/8 h long-day phytotron (21/19 °C).

### Induction of PX formation

For long-term microtubule imaging, 3-day-old dark-grown seedlings were transferred from ½ MS, 1% sucrose plates to plates containing an additional 10 µM dexamethasone (DEX). The unwrapped plates were kept in the same phytotron for 6–24 h after which seedlings were transferred to a microscope slide for imaging. The stages of PX formation were classified according to Watanabe et al.^9,23^. To quantify the progression of PX formation, we assigned weights of *w*_early_ = 1, *w*_mid_ = 2 and *w*_late_ = 3, respectively, to early-, mid- and late-stage cells, and used those to calculate the average stage.

### Cell wall analysis

Seeds of wild-type, VND7 and the EV control plants were grown under constant shaking at 100 rounds per minute in ½ MS medium supplemented with 1% sucrose at pH 5.7 in the dark. After 5 days, the cultures were supplemented with a final 10 µM DEX and were collected after 2 more days in the dark. Seedlings were stored in 70% ethanol for 1 week. To extract cell wall material, seedlings were first air-dried overnight in a 60 °C oven. The dry material was then frozen in liquid nitrogen and homogenized to a fine powder using metal balls and an oscillating mill (1 min at 25 Hz) from Retsch (Haan, Germany). Cell wall powder was washed with pure ethanol and centrifuged at 16,000 × *g* for 10 min. Subsequently, the pellet was resuspended in a 1 : 1 methanol : chloroform mixture and centrifuged again at 16,000 × *g* for 10 min. Lastly, the pellet was resuspended in pure acetone and centrifuged again at 16,000 × *g* for 10 min. After that, the cell wall pellet was air-dried overnight. The dry, insoluble part of the cell wall material was weighed to ~700–800 µg per 2 mL screw-cap tube. Subsequently, 250 µL of 2 M trifluoracetic acid was added to the tubes and incubated for 1 h at 121 °C. Afterwards, the tubes were supplemented with 300 µL of 2-Propanol and left for evaporation under a steady air flow at 40 °C. This step was repeated two times before the tubes were supplemented with 300 µl distilled water, thoroughly vortexed and centrifuged at 16,000 × *g* for 15 min. The pellet was further used to determine the amount of crystalline cellulose using the Updegraff method^50^.

### Microscopy and imaging

Fluorescence imaging was performed using a spinning disc microscope consisting of a CSU-X1 spinning disk head (Yokogawa) attached to an Eclipse TI inverted microscope (Nikon) equipped with a perfect focus system, an Evolve CCD camera (Photometrics), a Plan Apo ×100/1.4 NA oil-immersion objective and a ×1.2 lens between the spinning disk unit and the camera. YFP (yellow fluorescent protein) and GFP-labelled proteins were excited using a 491 nm solid-state laser and detected using a 525 nm filter with a 50 nm band pass (525/50). mCHERRY-labelled proteins, propidium iodide (PI) and DirectRed23 (both from Sigma) were excited using a 561 nm solid-state laser and detected using a 630/75 detection filter. All filters were purchased from Chroma. Data acquisition was performed using Metamorph software (Molecular Devices, Meta Imaging Series 7.7). Typical image acquisition settings were 1× gain, 300 EM gain, with exposure times of 100–300 ms for microtubules, 500–800 ms for GCP3-GFP and KTN1-GFP, and 500–2000 ms for PI and DirectRed23.

For imaging, seedlings were transferred to a cover slip (thickness #1.5) and covered by placing a 1 mm-thick 1% agarose (w/v) pad on top of the seedling as described by^12^. Briefly, a 22 × 30 mm^2^ cover slip was fixed centrally over a 15 mm-wide hole of an aluminium sample holder by means of vacuum grease. To assure proper attachment, the cover slip was gently pressed against the metal holder. The sample holder was turned upside down and up to three seedlings were typically placed. The seedlings were then covered with an ~1 mm-thick layer of agarose to which ~10 µL of sterile water was applied to provide a moist environment during imaging.

### Optimized sample preparation for long-term imaging

To image for extended periods of time (>4 h) we developed further the above described approach. We made sure that the cover slip was attached to the metal holder in a water-tight fashion by using more vacuum grease than previously done. After we placed the seedlings and covered them with the agarose pad, we entirely flooded the sample chamber and placed a second 22 × 30 mm^2^ cover slip on top to prevent exposure to room air. This created a closed cavity which was inert against evaporation for more than 24 h. We furthermore developed a journal that controlled the image acquisition of the Metamorph software (available upon request). We had to equip our spinning disk microscope with a motorized stage (from ASI) to allow computer-controlled stage movements. The journal records the position of up to six seedlings on the sample and runs a time-lapse at each of those sample positions. The length and time interval of each time lapse, as well as the total length of the continuous recording can be adjusted by the experimenter.

### Staining of hypocotyl cell walls and xylem secondary walls

To stain the walls of hypocotyl cells, seedlings were either transferred to 0.5 µg mL^−1^ PI in water for 10 min followed by a 5 min wash step in sterile water or to 0.02% DirectRed23 in water for 30 min followed by exchange of the medium with sterile water. Staining of cleared roots with basic Fuchsin was performed according to Mähönen et al.^51^. Briefly, the seedlings were cleared using 55 °C warm, acidified methanol and basic ethanol, stained using 0.01% basic Fuchsin and de-stained in a series of ethanol dilutions.

### Image analysis

Images were processed using Fiji software^52^ with the help of publicly available plug-ins such as MultiStackReg, Bleach Correction, Multiple Kymograph and Walking Average. The anisotropy of the cortical microtubules was determined using the Fibril Tool plug-in^28^, where an anisotropy value close to zero indicates a poorly aligned array (isotropic case) and a value close to one indicates an arrays with near perfect alignment (anisotropic case). The periodogram analysis was performed using a Matlab-based program, which converted intensity line scans along the growth axis of the cell into PSD using the periodogram function. The PSD was plotted against the inter-band spacing, derived from taking the inverse of the wavenumber. The peak of the PSD was identified as the mean spacing between microtubule bands.

### Measurement of microtubule dynamics parameters

Microtubule dynamics were measured according to Schneider et al.^30^. Briefly, we used a semi-automatic custom-made Fiji macro using the image difference function (Multi Kymograph/Stack Difference) to extract growing (positive differences) and shrinking (negative differences) microtubule ends comparable to the approach taken by Lindeboom et al.^12^. The macro extracts the positions of individual dynamic microtubule ends and thereby helps in identifying and counting the number of catastrophe and rescue events in a time-lapse recording. From these numbers we determined the overall catastrophe and rescue rate, *r*_cat_ and *r*_res_, respectively. The velocity of growing and shrinking microtubule ends was analysed using a high-throughput and randomized approach based on the kymograph analysis plug-in of the free software FIESTA^53^.

### Statistics and reproducibility

For the representative images shown, we repeated each experiment the following times (number of cells = *n*, number of seedlings = *N, r* = replicates, convention [*n*, *N, r*]): Fig. 1a: [3,4,6], Fig. 1f: [5,20] (only 4 cells were suitable for the analysis of microtubule rearrangements from beginning to end); Fig. 3a, b: [3,5,11] non-induced, [3,4,20] induced; Fig. 4a–d: [5,6,11] non-induced, [6,25,40] early induced, [6,17,25] mid induced, [2,6] late induced, Fig. 4g: [1,10,27] VND7, [1,14,28] *ktn1-2* VND7, Fig. 4h–k: [1,5,50] for wild-type and *ktn1-2*; Supplementary Fig. 1b: [1,6], Supplementary Fig. 1d: [3,4,12], Supplementary Fig. 1f: [1,4], Supplementary Fig. 2a: [977, 45, 1], Supplementary Fig. 4a: see Fig. 1f, Supplementary Fig. 5a: see Fig. 1f, Supplementary Fig. 6a: see Fig. 1f, Supplementary Fig. 7a: see Fig. 1f, Supplementary Fig. 12a: [1,4], Supplementary Fig. 15d: [563, 6, 1] wild type, [422, 6, 1] *ktn1-2*.

### Statistical analysis

Measured quantities were plotted using the Plots-Of-Data^54^ and PlotTwist^55^ web apps. The applied statistical tests were performed using Welch’s unpaired, two-sided *t*-test via a free online tool provided on the GraphPad website (https://www.graphpad.com/quickcalcs/ttest1.cfm). The 95% confidence intervals were provided by the PlotsOfData and PlotTwist web apps. Throughout the manuscript we used the following asterisk convention: *p < 0.05, **p < 0.005 and ***p < 0.0005. If not stated otherwise, we used Welch’s unpaired, two-sided *t*-test.

### Microtubule simulations

All simulations were performed with an extended version of the Cortical Sim software^32^. Please see the Supplementary Note 1 on how the software was extended and explanation for settings and parameters.

### Reporting summary

Further information on research design is available in the Nature Research Reporting Summary linked to this article.

## Supplementary information

Supplementary Information

Descriptions of Additional Supplementary Files

Supplementary Movie 1

Supplementary Movie 2

Supplementary Movie 3

Supplementary Movie 4

Supplementary Movie 5

Supplementary Movie 6

Supplementary Movie 7

Supplementary Movie 8

Supplementary Movie 9

Supplementary Data 1

Reporting Summary

## Data Availability

All imaging and simulation data sets are available upon request from the first author R.S. and corresponding author E.E.D., respectively. Source data are provided with this paper.

## Source data

Source Data

## Code availability

The computer simulation code is available upon request from corresponding author E.E.D.
