# Supplementary material for: Long-term single-cell imaging and simulations of microtubules reveal principles behind wall patterning during proto-xylem development
Source: Nat Commun. 2021 Jan 28;12:669. doi: 10.1038/s41467-021-20894-1 (PMC7843992; doi:10.1038/s41467-021-20894-1)
Supplement: Supplementary file 1 — Supplementary Information [file 41467_2021_20894_MOESM1_ESM.pdf]

Supplementary Figures

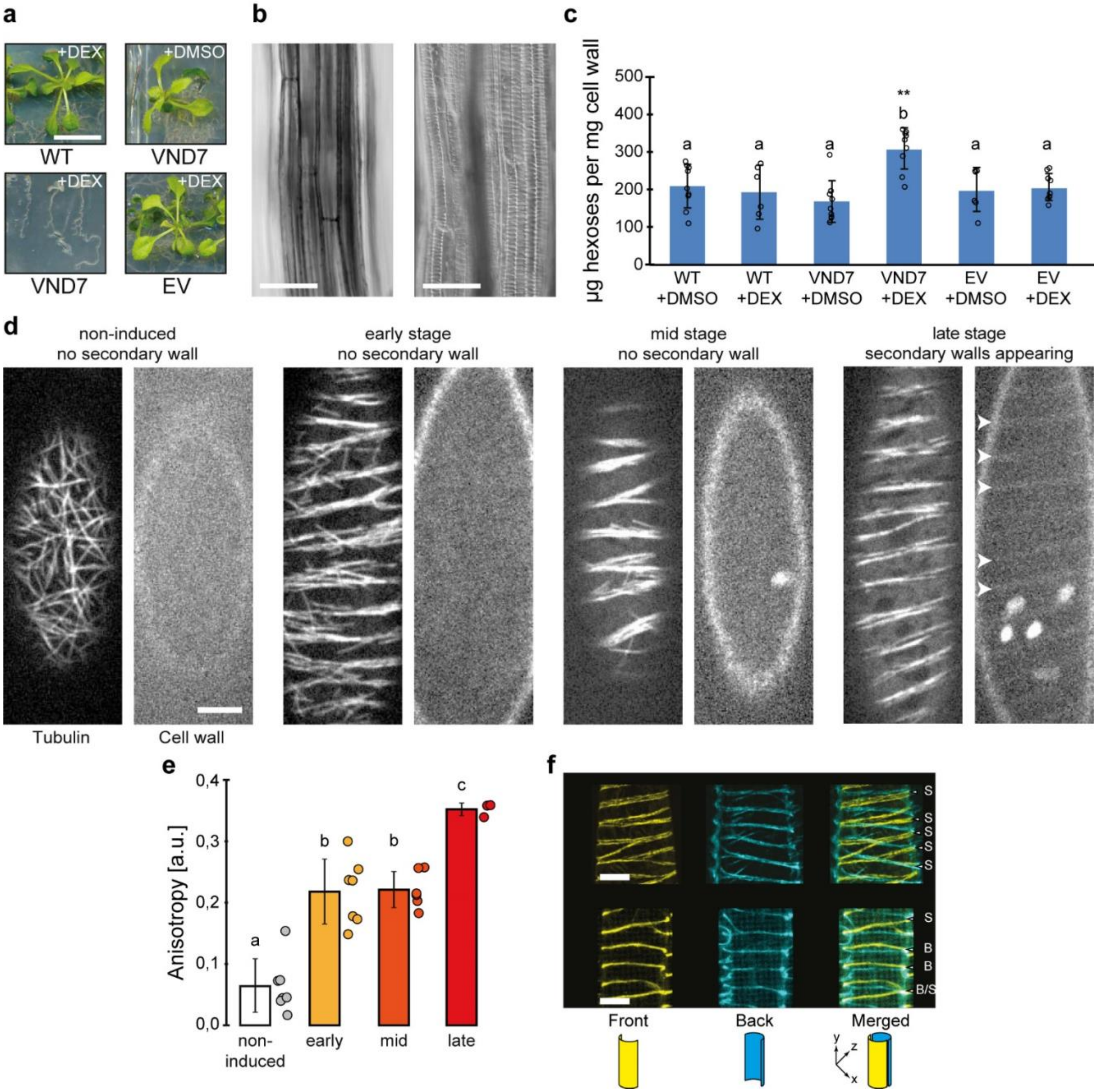

Supplementary Fig. 1 | The VND7-inducible system drives secondary wall deposition and cortical microtubule re-orientations in hypocotyl cells.

a 10-day old light-grown wild-type, VND7 and empty-vector (EV) seedlings treated with 10 µM DEX and DMSO, respectively, for 72 hours. Scale bar = 1 cm. **b** Differential interference contrast images of 10-day old dark-grown hypocotyls of wild type (left) and VND7 (right). Scale bar = 100 µm. **c** VND7-induced seedlings contain significantly more hexose sugars in their cell walls (309 ± 55 µg per mg cell wall material) compared to wild-type (208 ± 58 µg) and EV seedlings (199 ± 59 µg; mean ± sd, from 3 biological replicates with 3 technical replicates each. $p = 0.002$, Welch's unpaired, two-sided *t*-test). Groups assigned by statistical differences ($p < 0.05$ between groups). Dots depict hexose sugar contents of individual seedlings in each sample. **d** Representative stages of the VND7-driven secondary wall program: microtubule rearrangements precede the deposition of secondary walls (arrows heads, stained with Direct Red 23, see Methods). Scale bar = 5 µm. **e** The anisotropy of the microtubule array increased 5-fold from an initial 0.06 ± 0.04 (mean ± s.d., 7 cells from 5 seedlings) in non-induced cells to a final 0.35 ± 0.01 for cells in the late stage of proto-xylem formation. Groups assigned by statistical differences

based on Welch's unpaired, two-sided *t*-test ($p < 0.05$ between groups). Dots depict anisotropy measurements of 7, 7, 6, and 3 seedlings for non-induced, and induced early, mid, and late stages of proto-xylem differentiation. **f** Microtubule bands emerge across the entire cylindrical cell surface to form continuous spirals (labelled 'S', top) and parallel bands (labelled 'B', bottom). Microtubules in the outer and inner half of the cell (see illustrations below fluorescence images) labelled yellow (left) and cyan (middle), respectively. The merged image (right) thus color-codes for microtubules on the front and backside of the cell revealing spirals and bands occurring on both cellular interfaces. Scale bars = 10 µm.

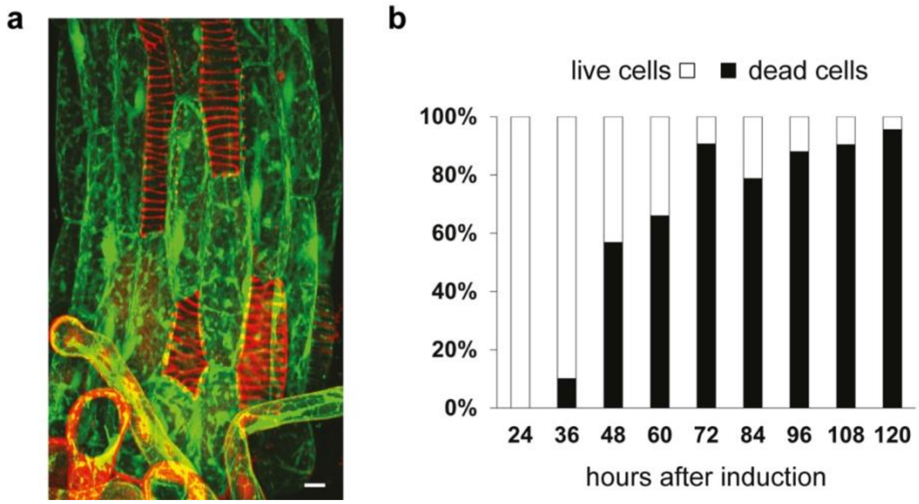

Supplementary Fig. 2 | The VND7-inducible system drives cell death in hypocotyl cells.
a VND7 induction leads to controlled cell death starting 24 hours after induction. Cell death is assessed via the life-stain fluorescein diacetate (FDA), which is converted by intracellular esterase activity in living cells resulting in fluorescein fluorescence (green). No esterase activity is present in dead cells resulting in no staining. Propidium iodide (PI, red) stains the secondary cell wall thickenings. Scale bar = 10 µm. **b** Time course of the fraction of cells undergoing programmed cell death after VND7 induction. Cells without FDA but with PI stain in (a) have completed the VND7 program and were scored as 'dead' cells (black). The majority of cells had died 120 hours after induction.

Supplementary Figures

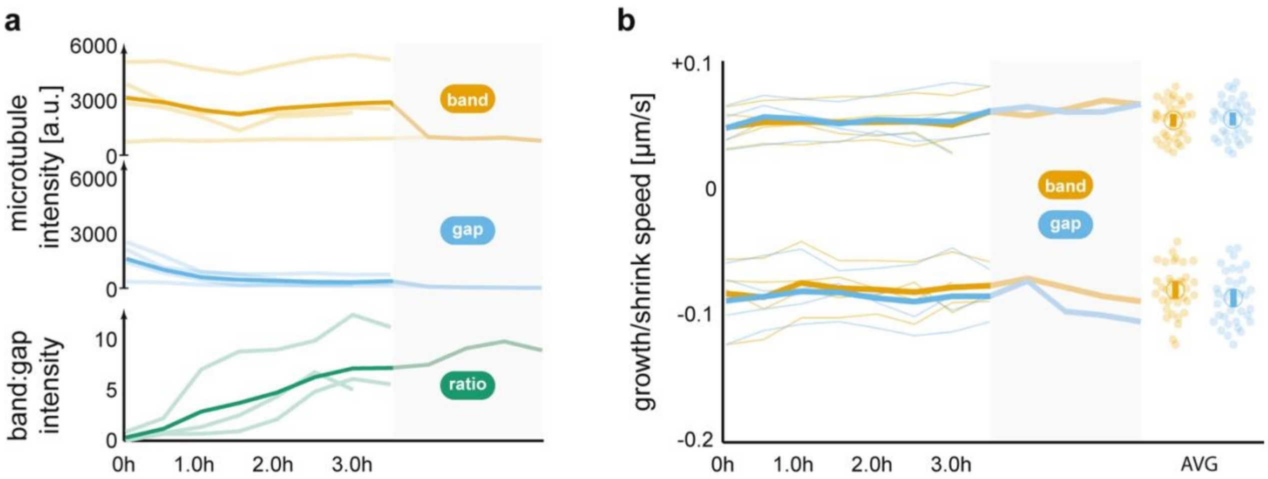

Supplementary Fig. 3 | The VND7-inducible system drives microtubule separation into bands and gaps in hypocotyl cells.

a Total microtubule intensity of four individual cells (pale lines, thick line = time average) divided into bands (orange) and gaps (blue), and the associated intensity ratio between bands and gaps (green). **b** Growth speed v^+ and shrink speed v^- of four individual cells (fine lines, thick line = time average) divided into band (orange) and gap regions (blue). Scatter plots: Data pooled from 35 time points of all four cells; means ± 95 % confidence intervals. No differences between growth and shrinkage speeds were found between bands and gaps. The shaded area in panel (a) and (b) indicate a period covered by only one dataset (Cell 4).

Supplementary Figures

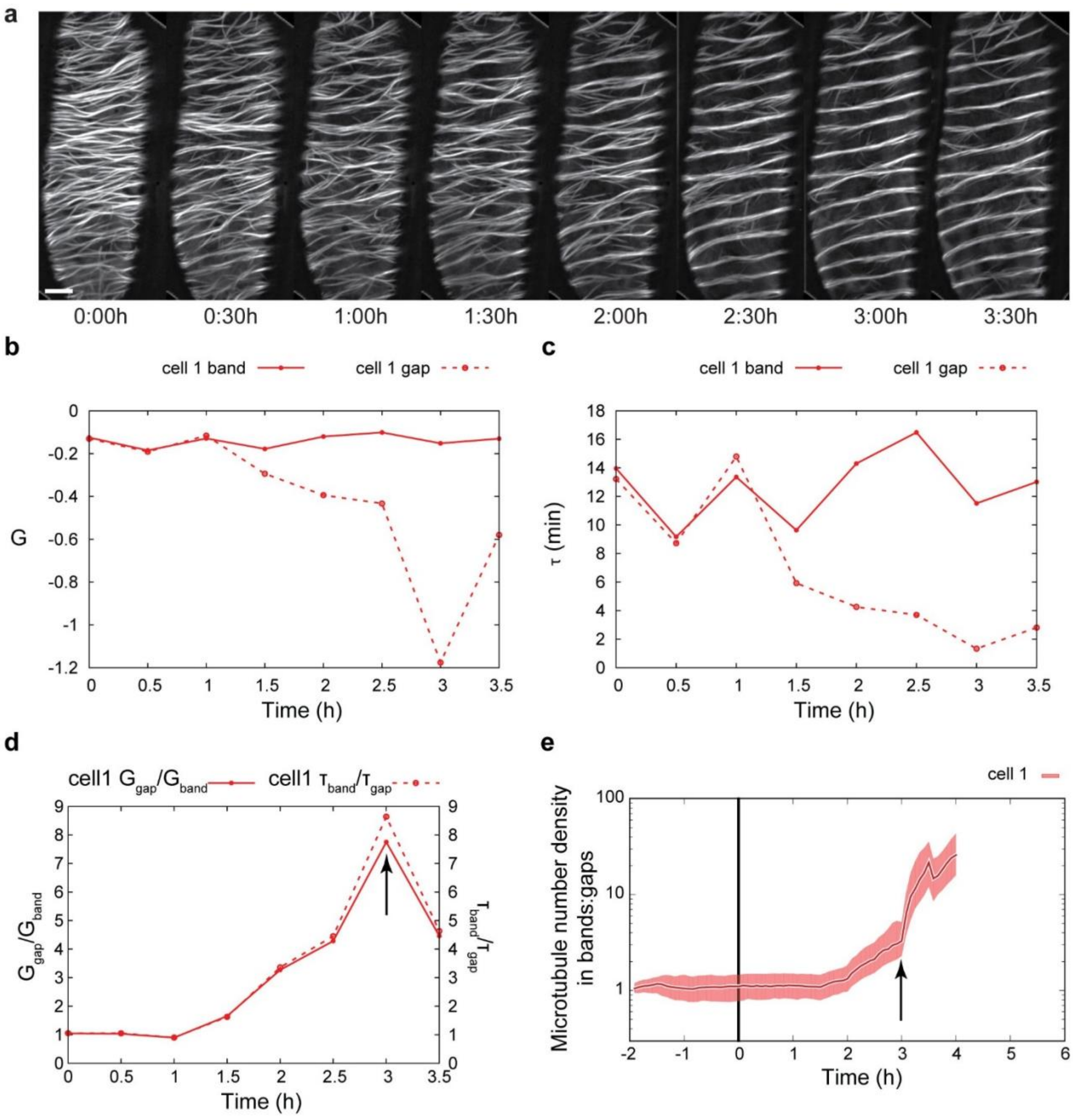

Supplementary Fig. 4 | Microtubule re-orientations during the VND7-driven secondary wall program for cell 1.

a Average projections of YFP-labelled microtubules. Scale bar = 5 µm. **b** Temporal development of the control parameter G in bands (solid line) and gaps (dashed line). This indicates that microtubules in the bands experience more interactions than microtubules in gaps. **c** Calculated microtubule lifetime τ in bands (solid line) and gaps (dashed line). Microtubules in the bands show longer lifetimes than in the gaps. **d-e** Temporal development of (d) the ratio of G_{gap}/G_{band} (solid line) and τ_{band}/τ_{gap} (dashed line) and (e) the degree of separation (= ratio of microtubules in bands versus gaps). Note that the time point of fastest separation (3h, arrow) coincides with peaks in G_{gap}/G_{band} and τ_{band}/τ_{gap} (arrow in d). This indicates that microtubule stability and average microtubule lifetime must be sufficiently different between bands and gaps for separation to occur. Data points in b-d calculated from data summarized in Supplementary Table 1. Line and margins in (e) represent median ± 16% and 84% percentiles from more than 100 individual simulation runs.

Supplementary Figures

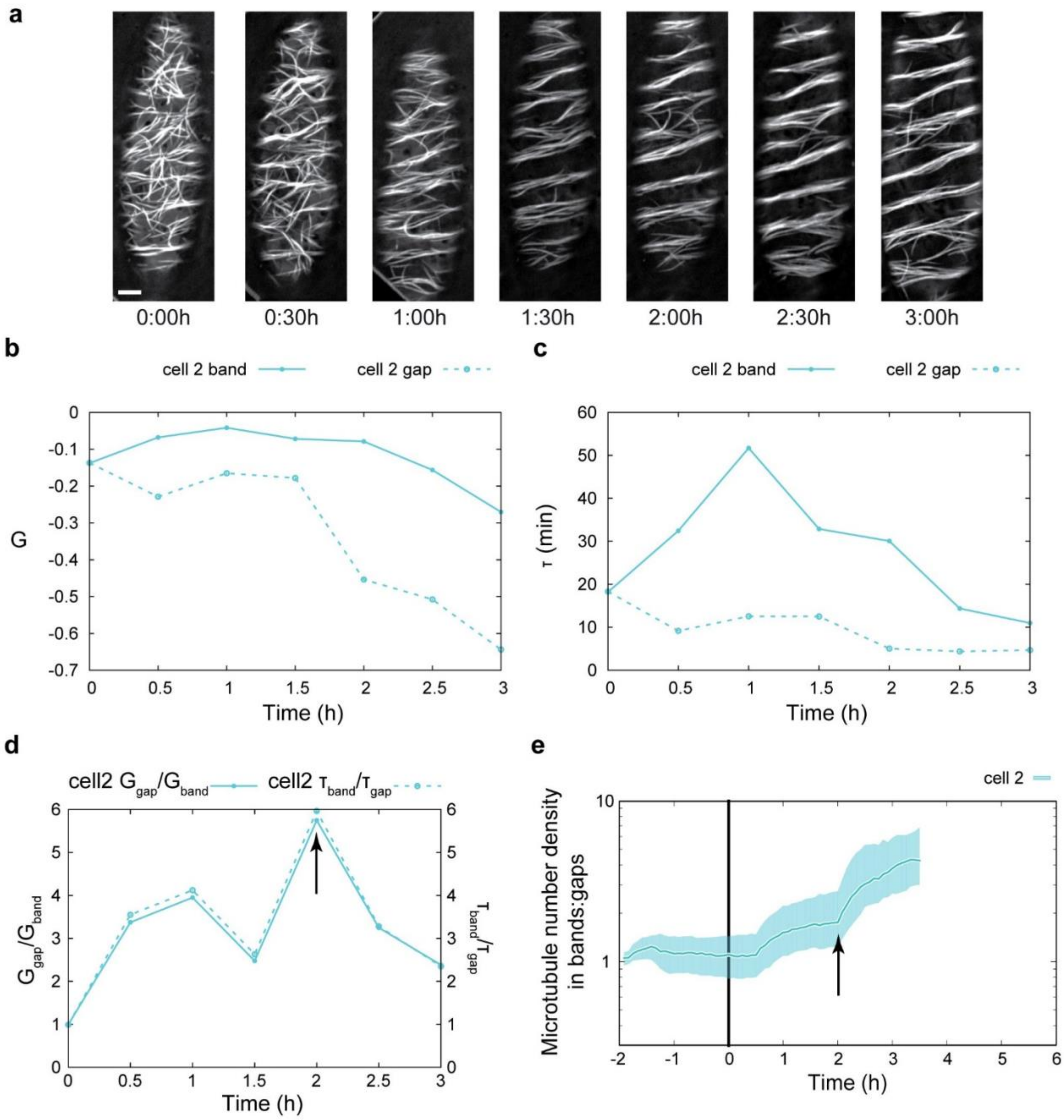

Supplementary Fig. 5 | Microtubule re-orientations during the VND7-driven secondary wall program for cell 2.

a Average projections of YFP-labelled microtubules. Scale bar = 5 µm. **b** Temporal development of the control parameter G in bands (solid line) and gaps (dashed line). This indicates that microtubules in the bands experience more interactions than microtubules in gaps. **c** Calculated microtubule lifetime τ in bands (solid line) and gaps (dashed line). Microtubules in the bands show longer lifetimes than in the gaps. **d-e** Temporal development of (d) the ratio of G_{gap}/G_{band} (solid line) and τ_{band}/τ_{gap} (dashed line) and (e) the degree of separation (= ratio of microtubules in bands versus gaps). Note that the time point of fastest separation (2h, arrows) coincides with peaks in G_{gap}/G_{band} and τ_{band}/τ_{gap} (arrow in d). This indicates that microtubule stability and average microtubule lifetime must be sufficiently different between bands and gaps for separation to occur. Data points in b-d calculated from data summarized in Supplementary Table 1. Line and margins in (e) represent median ± 16% and 84% percentiles from more than 100 individual simulation runs.

Supplementary Figures

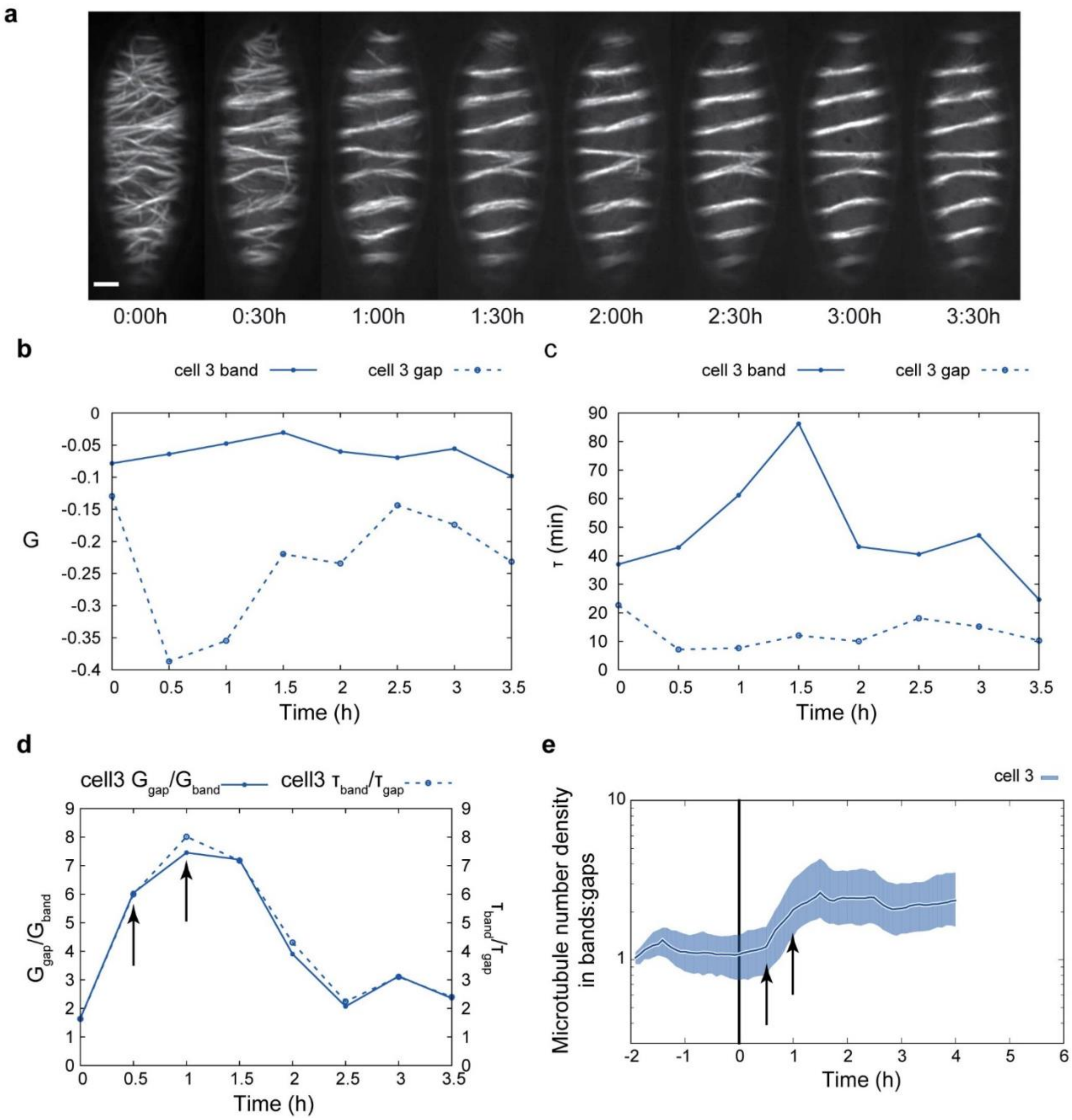

Supplementary Fig. 6 | Microtubule re-orientations during the VND7-driven secondary wall program for cell 3.

a Average projections of YFP-labelled microtubules. Scale bar = 5 µm. **b** Temporal development of the control parameter G in bands (solid line) and gaps (dashed line). This indicates that microtubules in the bands experience more interactions than microtubules in gaps. **c** Calculated microtubule lifetime τ in bands (solid line) and gaps (dashed line). Microtubules in the bands show longer lifetimes than in the gaps. **d-e** Temporal development of (d) the ratio of G_{gap}/G_{band} (solid line) and τ_{band}/τ_{gap} (dashed line) and (e) the degree of separation (= ratio of microtubules in bands versus gaps). Note that the time points of fastest separation (0.5 and 1h, arrows) coincides with peaks in G_{gap}/G_{band} and τ_{band}/τ_{gap} (arrows in d). This indicates that microtubule stability and average microtubule lifetime must be sufficiently different between bands and gaps for separation to occur. Data points in b-d calculated from data summarized in Supplementary Table 1. Line and margins in (e) represent median ± 16% and 84% percentiles from more than 100 individual simulation runs.

Supplementary Figures

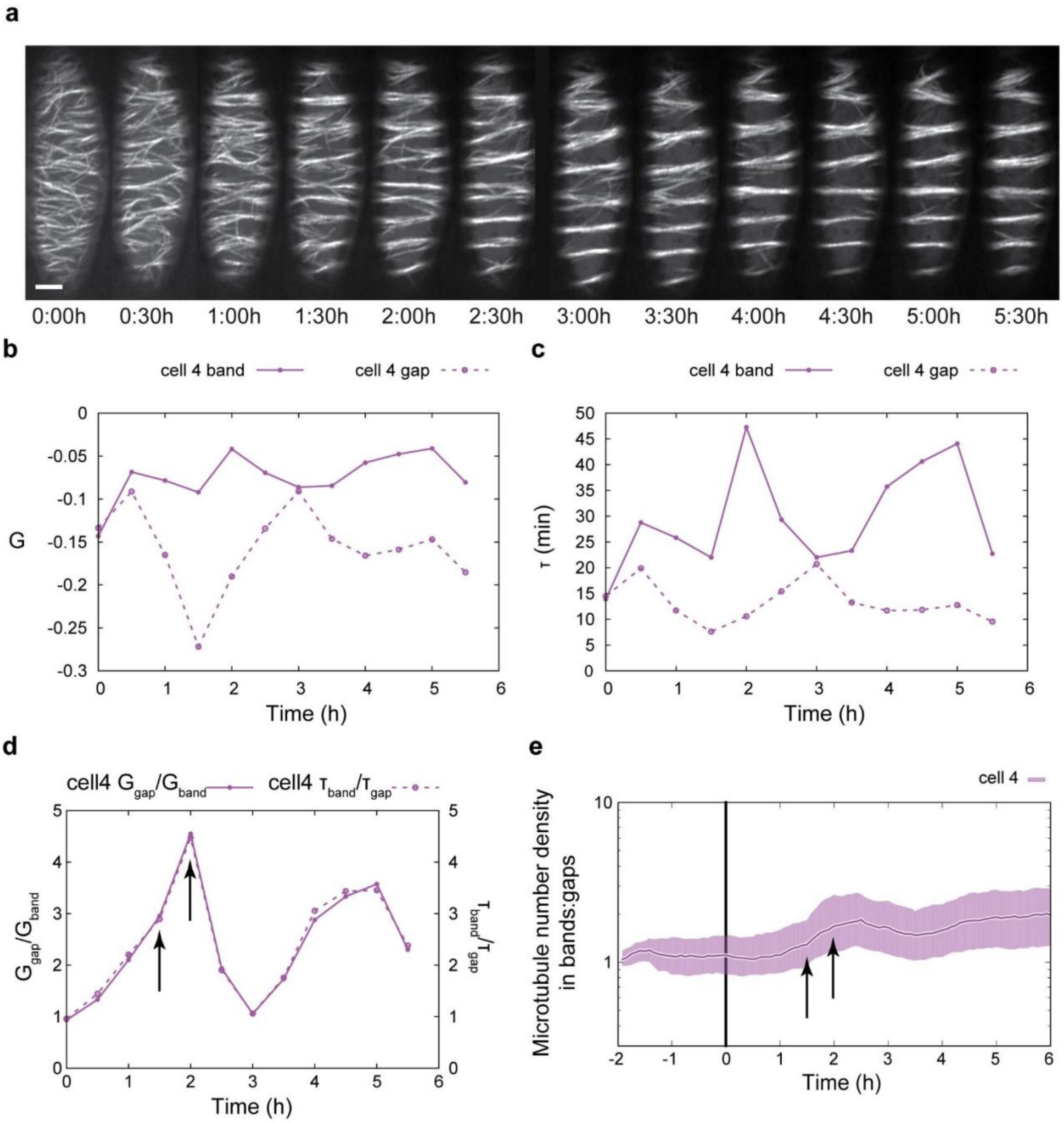

Supplementary Fig. 7 | Microtubule re-orientations during the VND7-driven secondary wall program for cell 4.

a Average projections of YFP-labelled microtubules. Scale bar = 5 µm. **b** Temporal development of the control parameter G in bands (solid line) and gaps (dashed line). This indicates that microtubules in the bands experience more interactions than microtubules in gaps. **c** Calculated microtubule lifetime τ in bands (solid line) and gaps (dashed line). Microtubules in the bands show longer lifetimes than in the gaps. **d-e** Temporal development of (d) the ratio of G_{gap}/G_{band} (solid line) and τ_{band}/τ_{gap} (dashed line) and (e) the degree of separation (= ratio of microtubules in bands versus gaps). Note that the time points of fastest separation (1.5 and 2h, arrows) coincides with peaks in G_{gap}/G_{band} and τ_{band}/τ_{gap} (arrows in d). This indicates that microtubule stability and average microtubule lifetime must be sufficiently different between bands and gaps for separation to occur. Data points in b-d calculated from data summarized in Supplementary Table 1. Line and margins in (e) represent median ± 16% and 84% percentiles from more than 100 individual simulation runs.

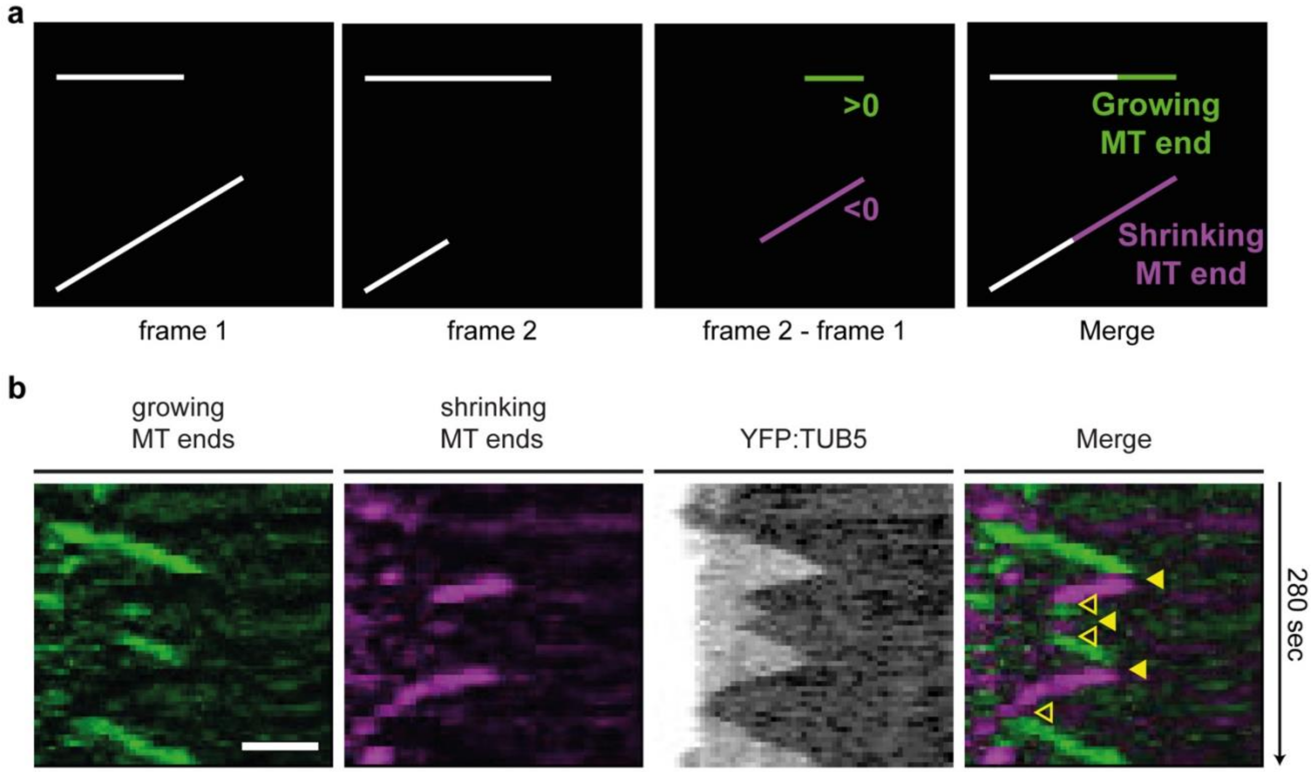

Supplementary Fig. 8 | Sequential-image subtraction to extract the dynamics of growing and shrinking microtubule ends in time-lapse recordings.

a Schematic showing the detection of growing (green) and shrinking (magenta) microtubules (white). A merged image (right panel) allows identification of the state, i.e. growing or shrinking, of a microtubule. **b** The dynamics of microtubule ends visualized using kymograph analysis of a merged image stack containing the growth and shrink information as illustrated in (a), allowing identification of locations where microtubules switched from growing to shrinking (filled arrow heads, termed ‘catastrophe’) and from shrinking to growing (empty arrow heads, termed ‘rescue’). Scale bar = 2 µm.

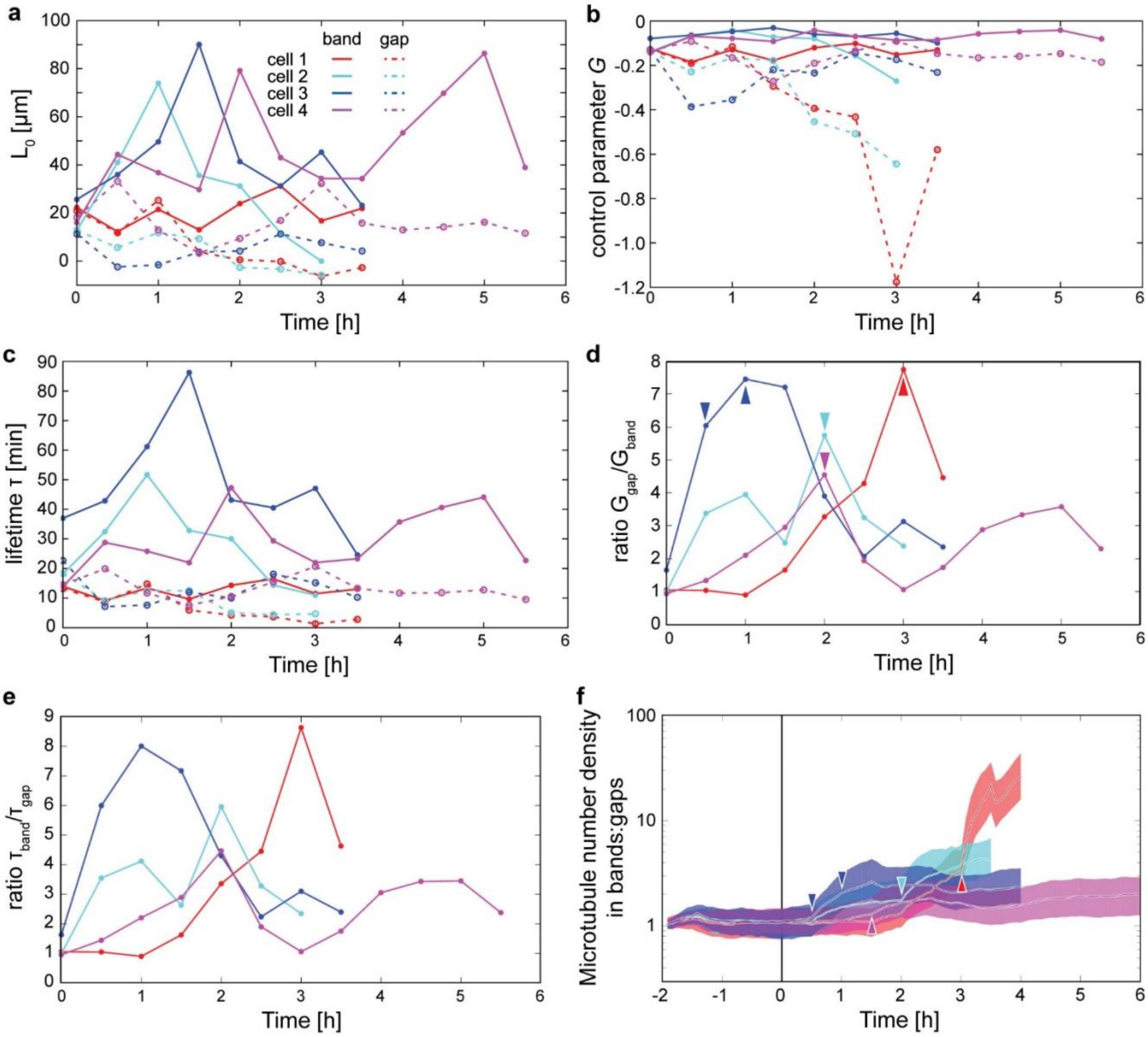

Supplementary Fig. 9 | Simulation of microtubule band formation using the measured microtubule dynamic instability parameters.

a Calculated length of non-interacting microtubules L_0 in bands and gaps for four long-term recordings (see legend for color-code). During band separation, the calculated average length of microtubules in the bands (solid lines) increased whereas the calculated average length of microtubules in the gaps (dashed lines) decreased. **b-e** Control parameter G (b) and average microtubule lifetime τ (c) in bands (solid) and gaps (dashed), and ratios of G_{gap}/G_{band} (d) and τ_{band}/τ_{gap} (e) for the four recordings. Note that the curves in a-e start at approximately similar values at T = 0h indicating that recording began before differences between bands and gaps became dominant. **f** Simulated degree of separation using the parameters obtained from the four analysed cells combining ten independent 'single-band' models to create 'reconstituted' 10-band structures. Time points of fastest separation: cell 1: 3 h, cell 2: 2 h, cell 3: 0.5 and 1 h, and cell 4: 1.5 and 2h (see coloured arrow heads). Line and margins in (f) represent median ± 16% and 84% percentiles from more than 100 individual simulation runs.

Supplementary Figures

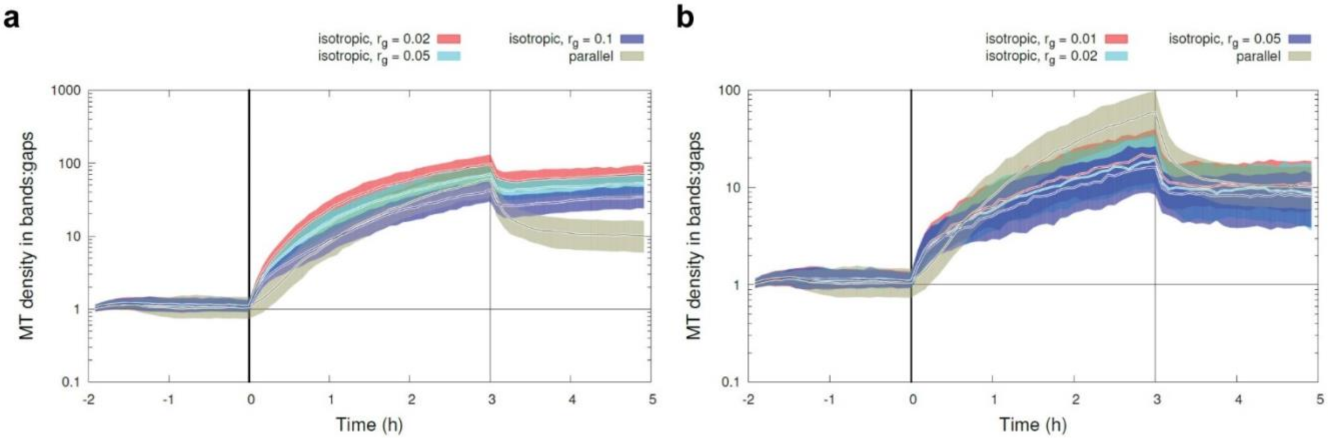

Supplementary Fig. 10 | Estimating the feedback strength of microtubule-bound nucleation.
a Comparing the default parallel nucleation with isotropic nucleation where rates in gaps were lowered and rates in bands were increased so that $r_{\mathrm{n,gap}} = r_{\mathrm{g}} \times r_{\mathrm{n,band}}$. The area-weighted average nucleation rate was the same as with parallel nucleation ($r_{n,parallel}$). The difference of nucleation rates in bands and gaps was 10-fold (blue, $r_{\mathrm{g}} = 0.10$), 20-fold (cyan, $r_{\mathrm{g}} = 0.05$), and 50-fold (red, $r_{\mathrm{g}} = 0.02$). Isotropic nucleation with a 20-fold rate difference between bands and gaps yields a similar degree of separation as parallel, microtubule-bound nucleation. **b** Comparing the default parallel nucleation with isotropic nucleation where only the rates in gaps were lowered and rates in the bands remained constant: $r_{n,band} = r_{n,parallel}$. The difference of nucleation rates in bands and gaps was 20-fold (blue, $r_{\mathrm{g}} = 0.05$), 50-fold (cyan, $r_{\mathrm{g}} = 0.02$), and 100-fold (red, $r_{\mathrm{g}} = 0.01$). Parallel, microtubule-bound nucleation always yielded larger degrees of separation. Note that, initially, microtubule-bound nucleation resulted in slower separation (a and b), because density differences build up over time. Line and margins represent median ± 16% and 84% percentiles from more than 400 individual simulation runs.

Supplementary Figures

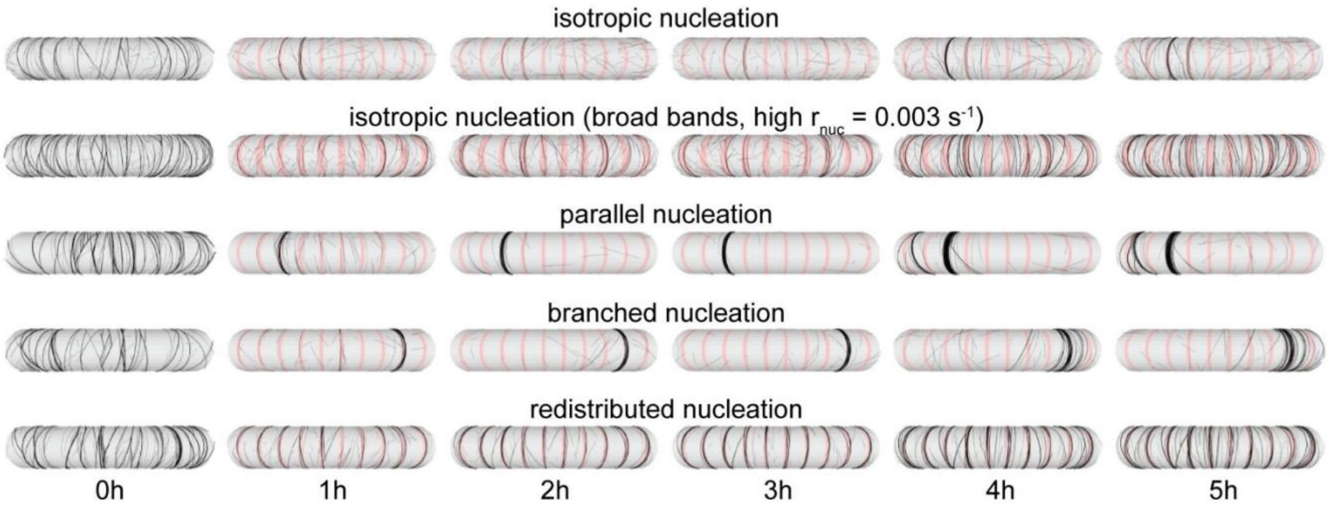

Supplementary Fig. 11 | Impact of nucleation mode on microtubule separation.
Randomly selected snapshots of representative simulations with the given nucleation mode over a time course of five hours.

Supplementary Figures

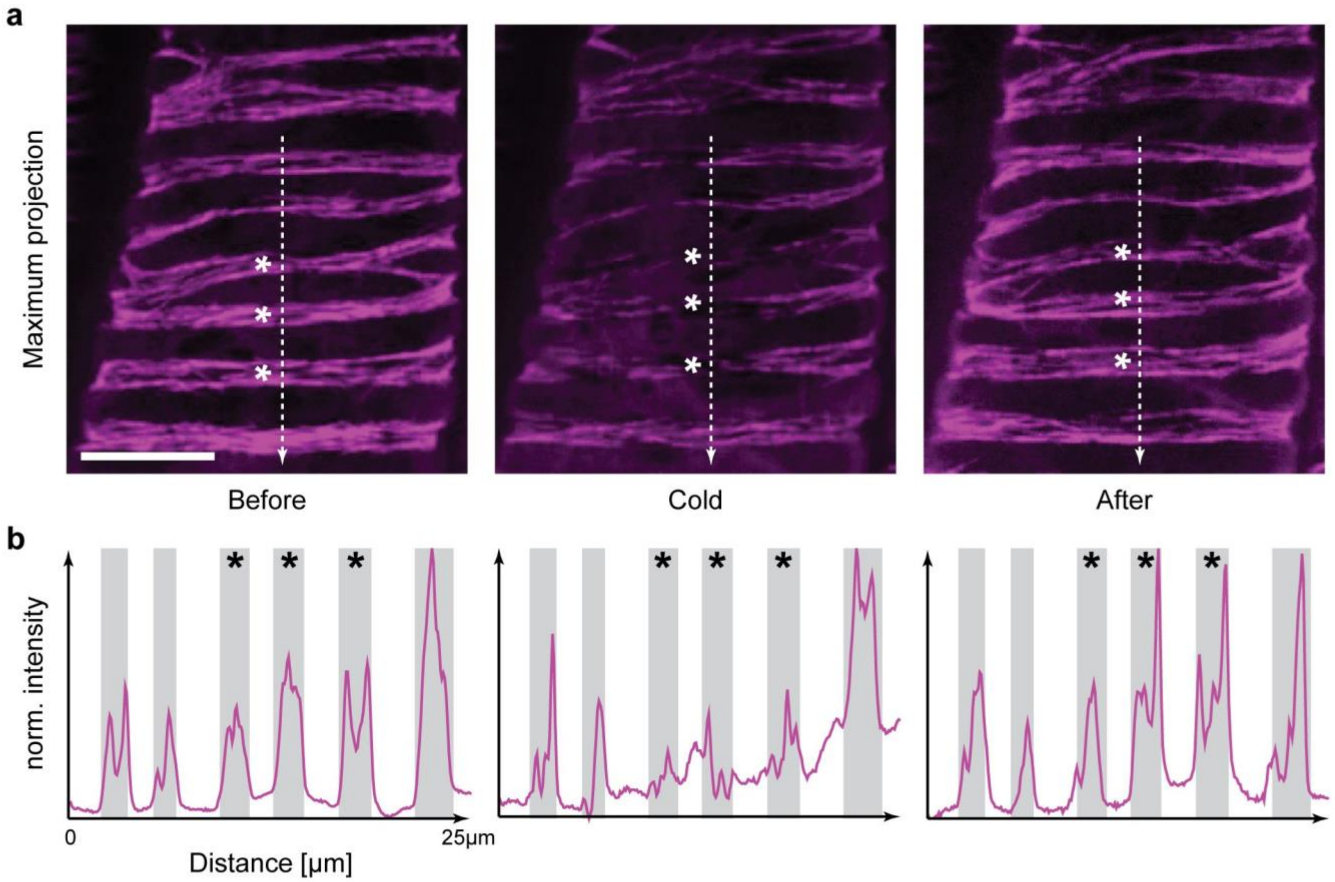

Supplementary Fig. 12 | Microtubules quickly re-populate bands after cold-induced microtubule depolymerization.

a Maximum projections of mCH-TUA5 labelled microtubules in mid stages of proto-xylem formation before (left), immediately after (middle), and 15 minutes after exposure to rapid cold. Microtubules depolymerize upon treatment (middle) as seen from lower microtubule decoration on bands (compare asterisk-labelled bands before and after), and a higher cytoplasmic background signal. After 15 minutes of recovery, the microtubule bands reformed to approx. the state before treatment. **b** Linescans of the normalized microtubule intensity along the dashed lines in (a) with bands indicated in grey. The microtubule bands highlighted with asterisks in (a) and (b) are identical. Scale bar = 10 µm.

Supplementary Figures

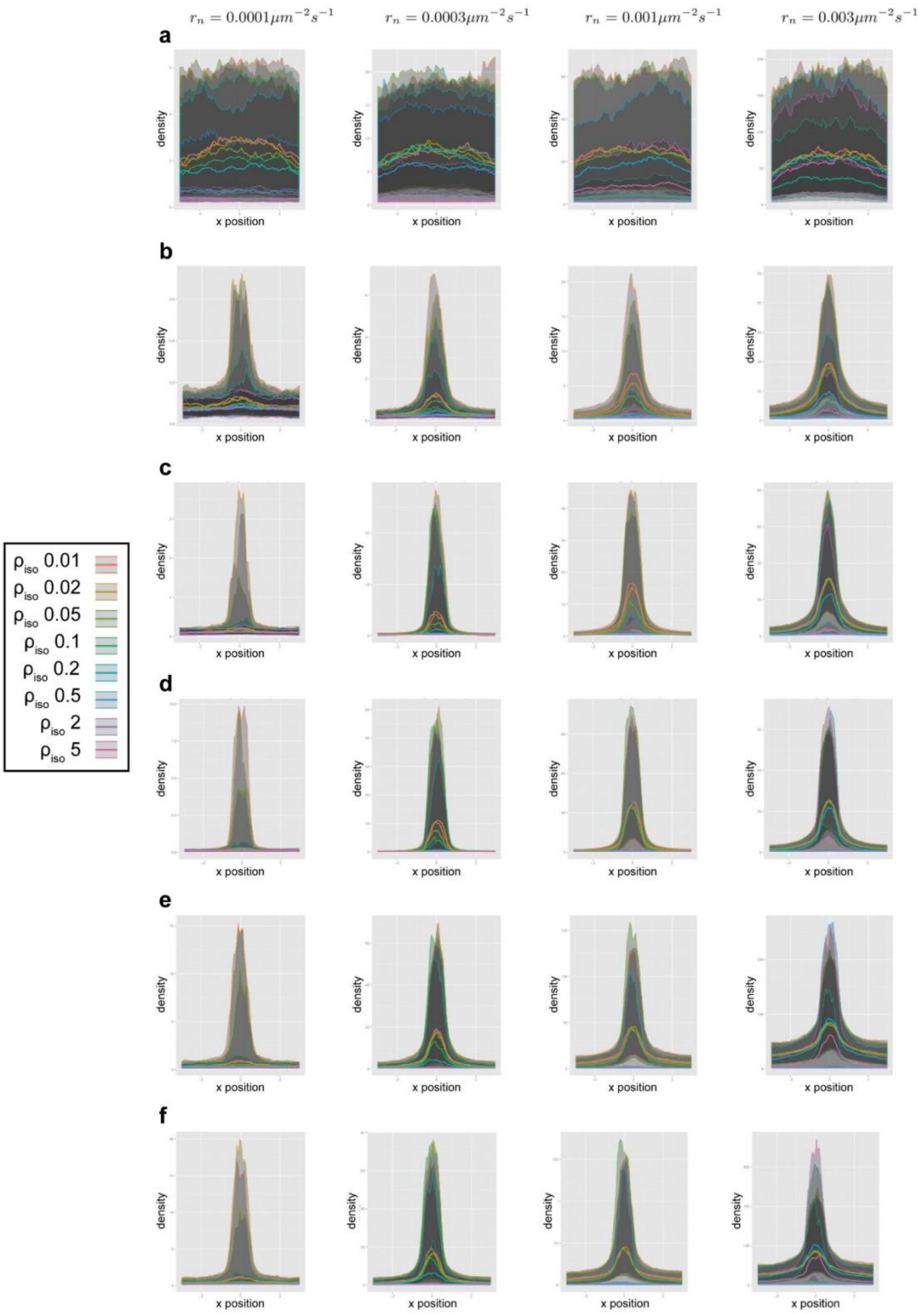

Supplementary Fig. 13 | Microtubule density histograms for multiple time stamps, variable ρ_{iso} (the density at which 50% of the nucleations occur from microtubules) per nucleation rate r_n.
a End of initiation phase (T = 0 h). **b** Separation phase (T = 1 h). **c** Separation phase (T = 2 h). **d** Separation phase (T = 3 h). **e** Middle of maintenance phase (T = 4 h). **f** End of maintenance phase (T = 5 h). All densities are in μm^{-1}. Similar results were obtained for further changing r_n and treadmilling speed v^t, as both impact total microtubule density.

Supplementary Figures

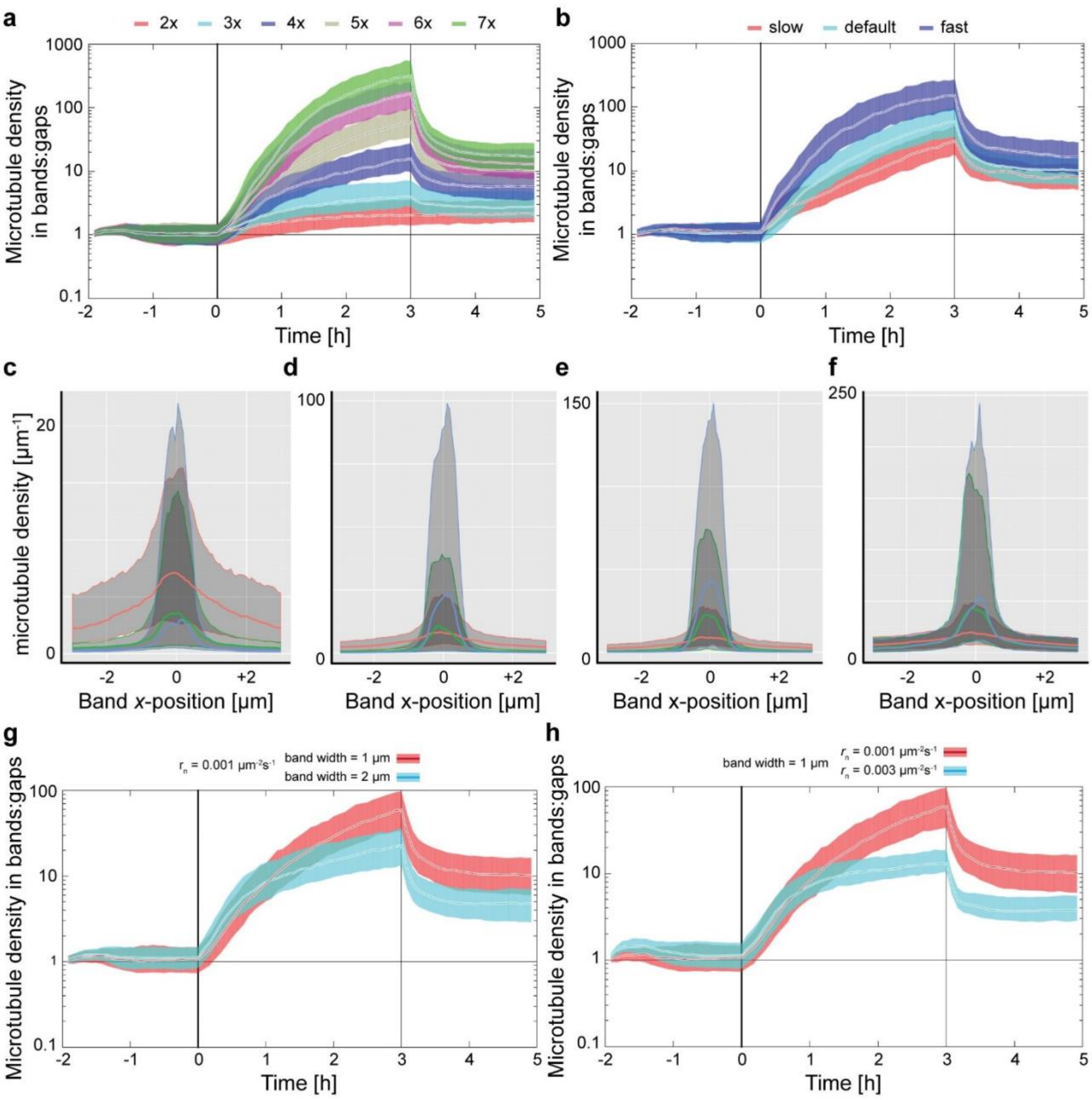

Supplementary Fig. 14 | Microtubule stability and turn-over are key determinants of band formation.

a Simulated degree of separation for six different G-ratios (separation strengths) resulting from two- to seven-fold difference in r_{cat} between bands and gaps. **b** Impact of different microtubule lifetimes τ at 5-fold separation strength. Microtubule lifetimes: 1.9 min (fast), 3.2 min (default), and 5.8 min (slow) in the gaps. Lines and margins in (a) and (b) represent median and 16% and 84% percentiles, respectively, from more than 100 individual simulation runs. **c-f** Histograms of microtubule density along the cell axis in single-banded models for three different separation strengths (3x red, 5x green, and 7x blue) and four subsequent time points: T = 1 h (c), T = 2 h (d), T = 3 h (e), and T = 4 h (f). Note the different scales. Thick lines and grey areas represent median and 16 % and 84 % percentiles, respectively. **g-h** Increasing the band width (g) from 1 to 2 µm or the nucleation rate (h) from 0.001 to 0.003 µm^{-2} s^{-1} had a negligible impact on the separation rate during the first hour of separation but affects saturation of the process. Lines and margins in (g) and (h) represent median and 16% and 84% percentiles, respectively, from more than 100 individual simulation runs.

Supplementary Figures

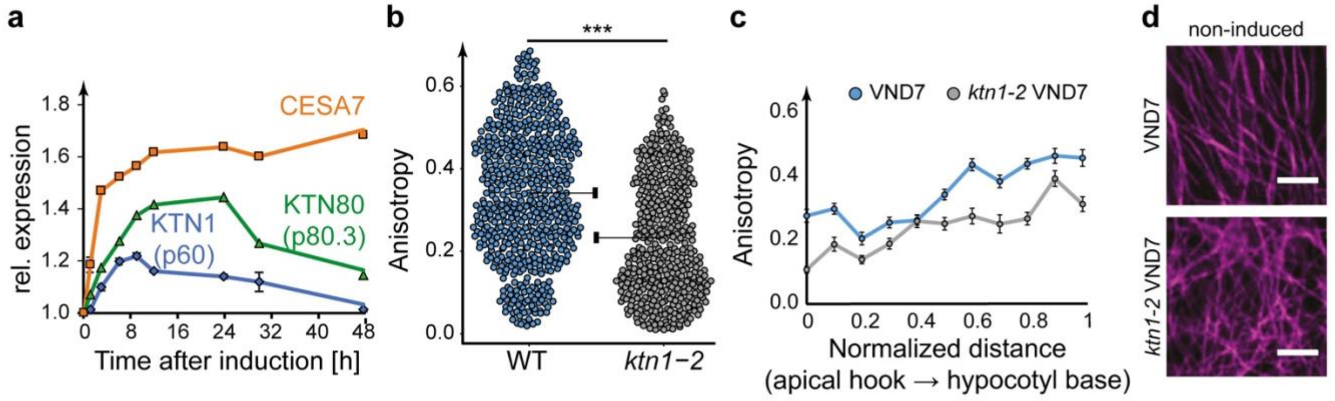

Supplementary Fig. 15 | KATANIN is upregulated in VND7 and loss-of-function microtubule arrays in the *ktn1-2* mutant show reduced anisotropy.

a Expression of the *KTN1* (p60 subunit) and *KTN80* (p80.3 isoform) in relation to secondary wall-specific *CESA7* over a time course of two days. Data normalized to expression levels in non-induced plants (data taken from Li et al., (2016b), mean ± sd, $n = 3$ replicates). **b** Wild-type seedlings show significantly higher microtubule anisotropies (0.34 ± 0.16, mean ± s.d., 563 cells from 7 dark-grown seedlings) compared to *ktn1-2* (0.23 ± 0.14, 422 cells, 6 seedlings, *** $p = 4.5 \times 10^{-27}$, Welch's unpaired, two-sided *t*-test) mutants. Scatter plots indicate mean (horizontal line) and 95% confidence intervals (vertical line). **c** Anisotropy of wild-type and *ktn1-2* microtubule arrays normalized to its position along the entire hypocotyl axis from the apical hook (= 0) to the base (= 1). The *ktn1-2* mutant shows consistently lower anisotropies than wild type along the entire hypocotyl length. Dots and error bars depict means ± sem. **d** Time-average projections of mCherry-TUA5 microtubules in non-induced wild-type (VND7, top) and *ktn1-2* seedlings (in the VND7 background, bottom). Microtubules in non-induced cells of wild-type (VND7) seedlings showed better co-alignment compared to *ktn1-2* mutants. Scale bar = 5 µm.

Supplementary Figures

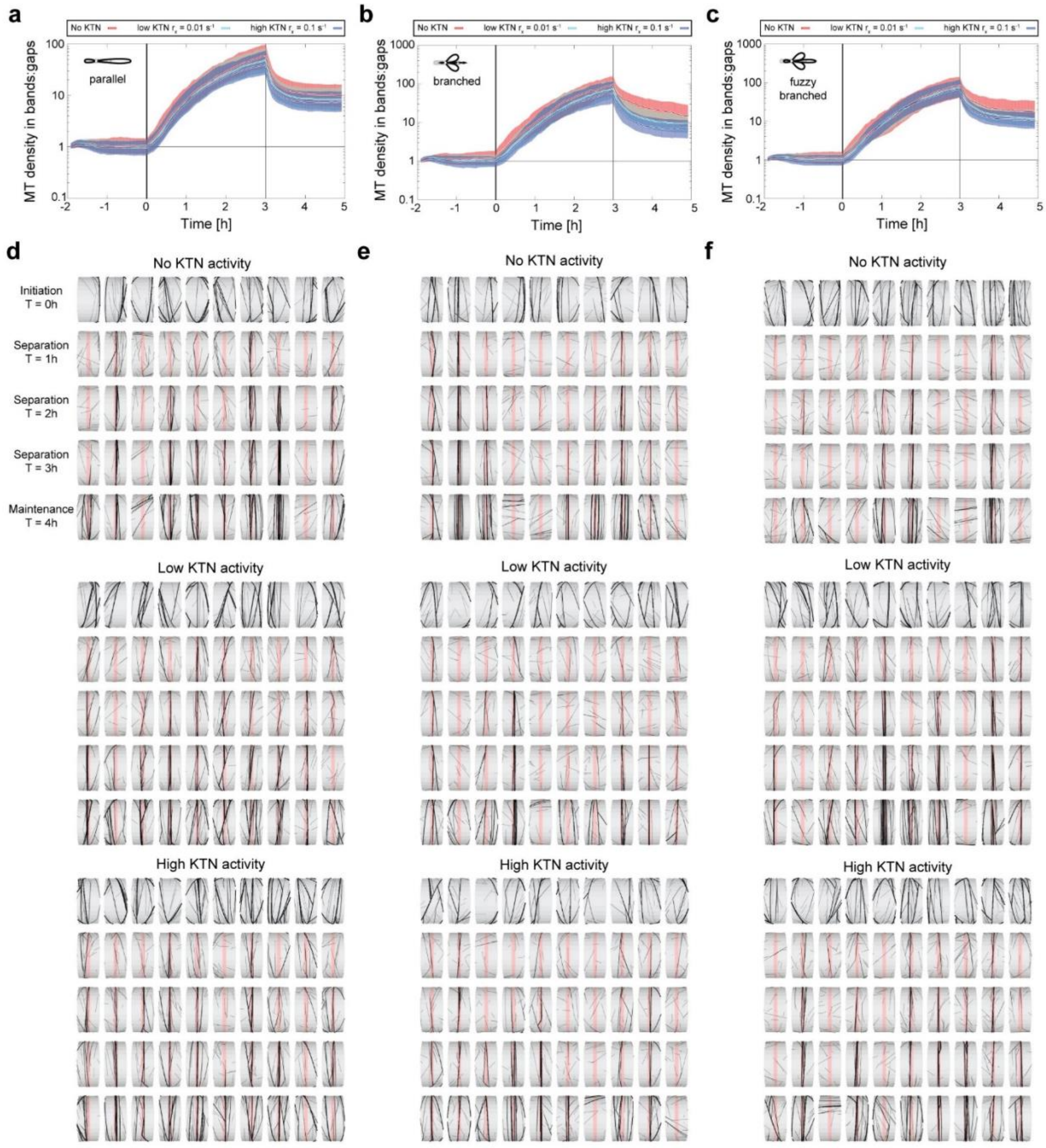

Supplementary Fig. 16 | Severing at crossovers has little impact on the separation process, independent of the type of nucleation mode.

a-c Degree of separation for parallel (a), branched (b), and fuzzy branched (c; similar to branched, except that small deviations are applied to all microtubule-based nucleations, including the (anti-)parallel ones) nucleation modes with zero severing rate $r_x = 0$ (red line), with low severing rate $r_x = 0.01\ s^{-1}$ per crossover (cyan), and with high $r_x = 0.1\ s^{-1}$ (blue). Lines and margins represent median and 16% and 84% percentiles, respectively, from more than 100 individual simulation runs. **d-f** Example snapshots of 'reconstituted' single-band models, visually selected for transverse orientation at T=0h (start of separation phase). The same structures are shown at 0, 1, 2, 3 and 4h. Left to right shows different nucleation modes: parallel (d), branched (e), and fuzzy branched (f). Top to bottom shows different severing rates for KTN. Top: no severing ($r_x = 0$), middle: low severing rate ($r_x = 0.01\ s^{-1}$) per crossover, bottom: high severing rate ($r_x = 0.1\ s^{-1}$) per crossover.

Supplementary Figures

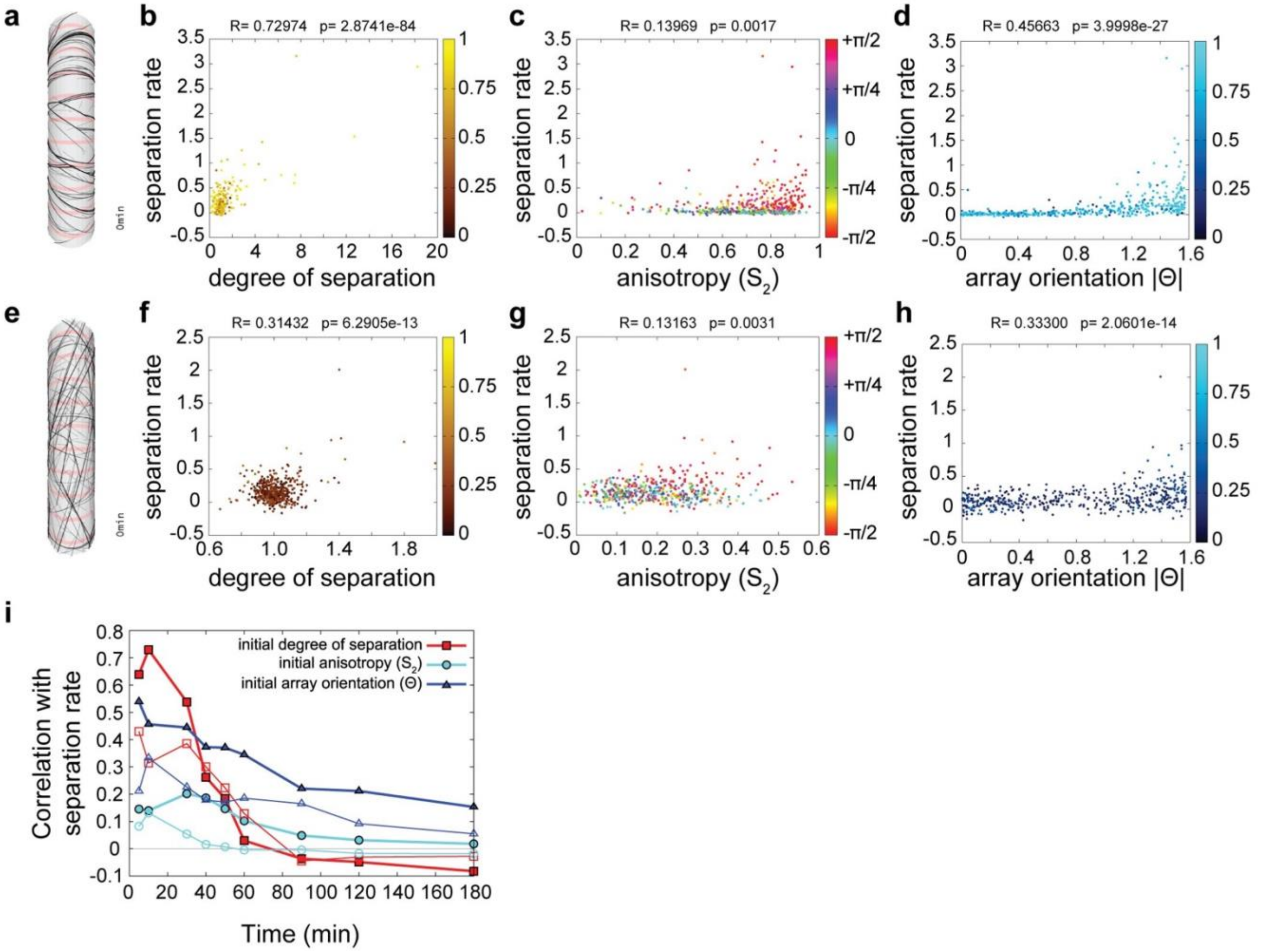

Supplementary Fig. 17 | Initial microtubule array orientation and anisotropy impact separation speed *in silico*.

a Example of an initial array state initiated with P_{cat} = 0.5. **b-d** Scatter plots of the separation rate at T = 10 min and the degree of separation (b), the anisotropy (S_2 parameter, c), and the array orientation Θ (d) at T = 0 min, for P_{cat} = 0.5 during the initiation phase. **e** Example of an initial array state initiated with P_{cat} = 0.05. **f-h** Scatter plots of the separation rate at T = 10 min and the degree of separation (b), the anisotropy (S_2 parameter, c), and the array orientation Θ (d) at T = 0 min, for P_{cat} = 0.05 during the initiation phase. All panels have a default P_{cat} of 0.5 during separation phase. Panels b,d,f,h: data points are coloured by the value of the anisotropy parameter S_2 as indicated. Panels c,g: data points are coloured by Θ value (in radians) as indicated. Red corresponds to a transverse array. **i** Correlations between separation rate since $T = 0$ and (i) the initial degree of separation (red), (ii) initial anisotropy (S_2 parameter, cyan), and (iii) initial array orientation (Θ, blue). All arrays are initiated with isotropic nucleation, followed by parallel nucleation during separation phase. Closed symbols: P_{cat} = 0.5 during entire simulation; open symbols: P_{cat} = 0.05 during initiation phase, and P_{cat} = 0.5 afterwards. Analysis is based on more than 500 'reconstituted' single-cell structures.

Supplementary Figures

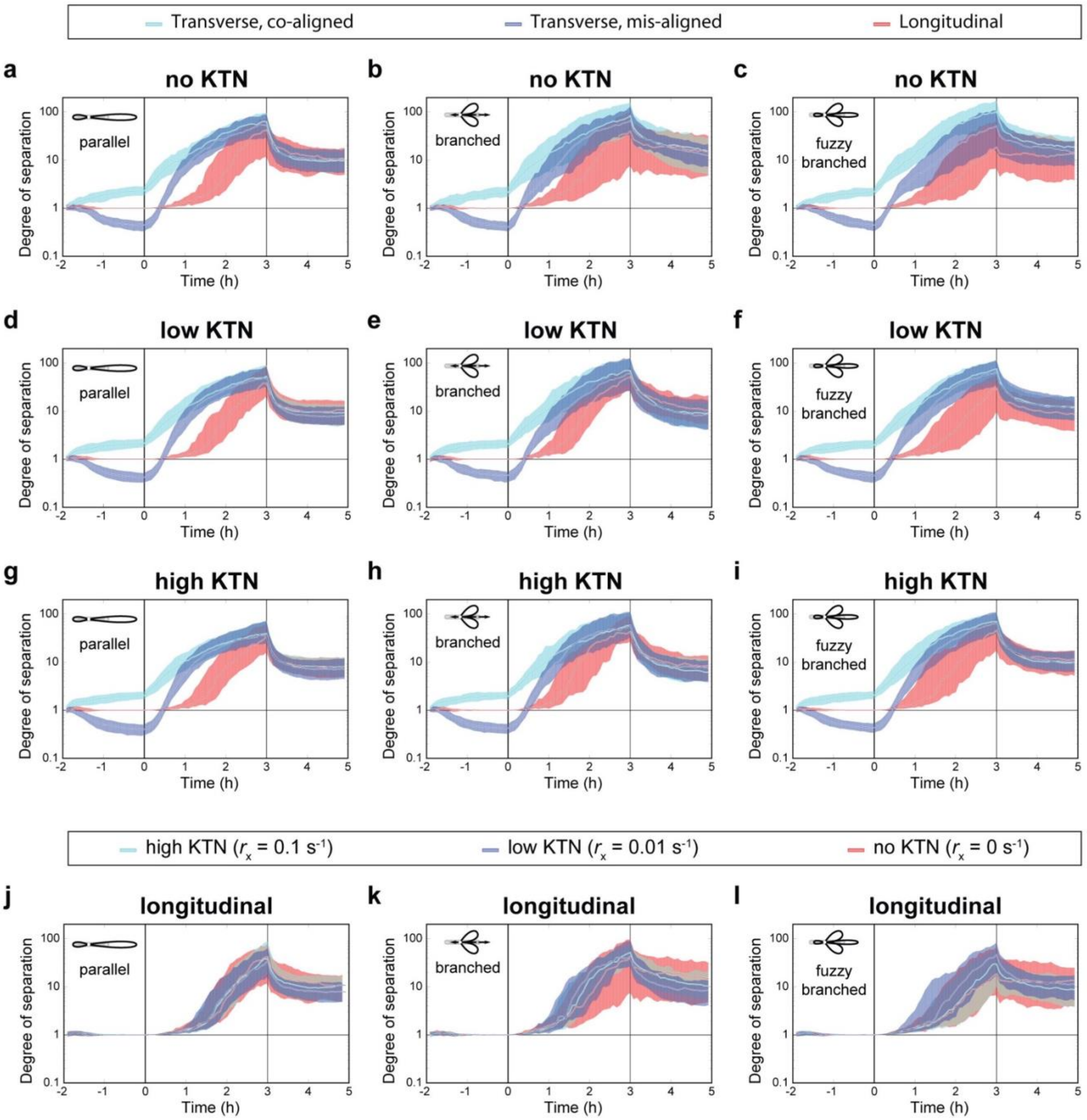

Supplementary Fig. 18 | Band separation is fastest for initially co-aligned transverse arrays and slowest for initially longitudinal arrays, independent of KTN activity and nucleation mode.
The initial microtubule arrays (selected at T = 0h) were sorted into three groups: i) transverse and co-aligned with band (cyan, array orientation angle between 90 ± 10 degrees and largest density in band), ii) transverse and mis-aligned with band (blue, array orientation angle between 90 ± 10 degrees and largest density > 1 µm away from the band), and iii) longitudinal (red, array orientation angle between 0 ± 10 degrees). **a-c** no KTN severing, **d-f** low KTN severing rate (r_x = 0.01 s^{-1}), **g-i** high KTN severing rate (r_x = 0.1 s^{-1}). Simulations assuming default parallel (a, d, g), or branched (b, e, h), and fuzzy branched (c, f, i) nucleation yield qualitatively similar behaviour: transverse co-aligned and mis-aligned microtubule arrays separate fastest and yield comparable degrees of separation. **j-l** separation of longitudinal arrays compared for parallel (j), branched (k), and fuzzy branched (l) for all KTN rates: no KTN severing (red), low KTN severing rate (cyan, r_x = 0.01 s^{-1}), and high KTN severing rate (blue,

r_x = 0.1 s^{-1}). A subtle quantitative effect of KTN on separation speed can be found for fuzzy branched nucleation (l) with high KTN severing rate separating longitudinal arrays slightly faster than low and no KTN severing rate. Fraction of simulations sorted in i) transverse and co-aligned with band (TC), ii) transverse and mis-aligned with band (TM), and iii) longitudinal (L) of all simulations performed: (a) TC: 26%, TM: 28%, L: 22%. (b) TC: 31%, TM: 23%, L: 21%. (c) TC: 26%, TM: 27%, L: 22%. (d) TC: 27%, TM: 30%, L: 18%. (e) TC: 29%, TM: 30%, L: 17%. (f) TC: 29%, TM: 29%, L: 17%. (g) TC: 28%, TM: 32%, L: 14%. (h) TC: 27%, TM: 30%, L: 15%. (i) TC: 27%, TM: 32%, L: 13%. (j) no: 22%, low: 18%, high: 14%. (k) no: 21%, low: 17%, high: 15%. (l) no: 22%, low: 17%, high: 13%. When selecting at the end of separation phase (T=3h), the fraction of longitudinal cells decreases with increasing severing activity: parallel: no: 1.5%, low: 1.3%, high: 1.0%; branched: no: 4.0%, low: 3.0%, high: 1.5%; fuzzy branched: no: 4.5%, low: 3.0%, high: 2.3%. This indicates that severing helps maintain/reach the most favourable array state. Lines and margins represent medians ± 16% and 84% percentiles from indicated fractions of more than 100 individual simulation runs.
