# Supplementary material for: Long-term single-cell imaging and simulations of microtubules reveal principles behind wall patterning during proto-xylem development
Source: Nat Commun. 2021 Jan 28;12:669. doi: 10.1038/s41467-021-20894-1 (PMC7843992; doi:10.1038/s41467-021-20894-1)
Supplement: Supplementary file 2 — Descriptions of Additional Supplementary Files [file 41467_2021_20894_MOESM2_ESM.pdf]

**Descriptions of Additional Supplementary Files**

**Supplementary Data 1**
**Description:** Parameter file examples.

**Supplementary Movie 1**
**Description:** Low-temporal resolution time-lapse recording of microtubule rearrangements during proto-xylem formation. Hypocotyl cell expressing mCH-TUA5 microtubules 11 hours after induction. Images recorded at 30 second intervals for > 4 hours. Scale bar = 2 µm.

**Supplementary Movie 2**
**Description:** High-temporal resolution time-lapse recording of microtubule rearrangements during proto-xylem formation – Cell 1. Hypocotyl cell expressing YFP-TUB microtubules. Images recorded at 5-second intervals every 30 minutes for approx. 3.5 hours. Scale bar = 10 µm.

**Supplementary Movie 3**
**Description:** High-temporal resolution time-lapse recording of microtubule rearrangements during proto-xylem formation – Cell 2. Hypocotyl cell expressing YFP-TUB microtubules. Images recorded at 5-second intervals every 30 minutes for approx. 3 hours. Scale bar = 10 µm.

**Supplementary Movie 4**
**Description:** High-temporal resolution time-lapse recording of microtubule rearrangements during proto-xylem formation – Cell. Hypocotyl cell expressing YFP-TUB microtubules. Images recorded at 5-second intervals every 30 minutes for approx. 3.5 hours. Scale bar = 10 µm.

**Supplementary Movie 5**
**Description:** High-temporal resolution time-lapse recording of microtubule rearrangements during proto-xylem formation – Cell 4. Hypocotyl cell expressing YFP-TUB microtubules. Images recorded at 5-second intervals every 30 minutes for approx. 5.5 hours. Scale bar = 10 µm.

**Supplementary Movie 6**
**Description:** GCP3 nucleating a new microtubule from an existing parent microtubule in non-induced cells. Hypocotyl cell expressing mCH-TUA5 microtubules (left) and GCP3-GFP (middle). The position of the microtubule plus-end is indicated by a white line moving with the growing tip, and the nucleating GCP3 is indicated by an arrowhead in merged images (right). Images recorded at 5-second intervals. Scale bar = 5 µm.

**Supplementary Movie 7**
**Description:** Example of highly variable emergence angles of newly polymerized microtubules and linear movements of GCP3s in induced cells. Hypocotyl cell expressing mCH-TUA5 microtubules (left) and GCP3-GFP (middle). The polymerizing microtubules are indicated by grey lines, and two nucleating GCP3s are indicated by arrowheads. Merged images are shown in the right panel. A microtubule that is growing into the gap shows significant angular swivelling until it forms a temporary (anti-parallel) bundle with another microtubule emerging from the neighbouring band. Images recorded at 5-second intervals. Scale bar = 5 µm.

**Supplementary Movie 8**

**Description:** Example of a severed microtubule being transported along a newly polymerized microtubule in anti-parallel orientation.

Hypocotyl cell expressing mCH-TUA5 microtubules (left) and GCP3-GFP (middle). The relevant microtubules are indicated by grey lines, and a nucleating GCP3 is indicated by an arrowhead. Merged images are shown in the right panel. One microtubule grows into the gap from the right and is subsequently severed (yellow line). The severed microtubule seems to flip over to the newly polymerized microtubule (forming an anti-parallel bundle) and is subsequently being transported away from the nucleating GCP3. Images recorded at 5-second intervals. Scale bar = 5 μm.

**Supplementary Movie 9**

**Description:** Example of an anti-parallel microtubule bundle exerting pulling action on a nucleating GCP3 leading to linear displacement.

Hypocotyl cell expressing mCH-TUA5 microtubules (left) and GCP3-GFP (middle). The relevant microtubules are indicated by grey lines, and the nucleating GCP3 is indicated by an arrowhead. Merged images are shown in the right panel. Two microtubules grow towards each other from opposing bands and form an anti-parallel bundle. The microtubule bundle seems to anchor to the lower microtubule band via a GCP3. This GCP3 is temporarily shifted into the gap region supposedly driven by relative microtubule sliding. Images recorded at 5-second intervals. Scale bar = 5 μm.
