# Supplementary material for: Long-term single-cell imaging and simulations of microtubules reveal principles behind wall patterning during proto-xylem development
Source: Nat Commun. 2021 Jan 28;12:669. doi: 10.1038/s41467-021-20894-1 (PMC7843992; doi:10.1038/s41467-021-20894-1)
Supplement: Supplementary file 13 — Reporting Summary [file 41467_2021_20894_MOESM13_ESM.pdf]

nature research

Corresponding author(s): Staffan Persson
NCOMMS-20-21933

Last updated by author(s): Dec 15, 2020

Reporting Summary

Nature Research wishes to improve the reproducibility of the work that we publish. This form provides structure for consistency and transparency in reporting. For further information on Nature Research policies, see our Editorial Policies and the Editorial Policy Checklist.

Statistics

For all statistical analyses, confirm that the following items are present in the figure legend, table legend, main text, or Methods section.

n/a	Confirmed	
☐	☒	The exact sample size (*n*) for each experimental group/condition, given as a discrete number and unit of measurement
☐	☒	A statement on whether measurements were taken from distinct samples or whether the same sample was measured repeatedly
☐	☒	The statistical test(s) used AND whether they are one- or two-sided<br>*Only common tests should be described solely by name; describe more complex techniques in the Methods section.*
☒	☐	A description of all covariates tested
☒	☐	A description of any assumptions or corrections, such as tests of normality and adjustment for multiple comparisons
☐	☒	A full description of the statistical parameters including central tendency (e.g. means) or other basic estimates (e.g. regression coefficient) AND variation (e.g. standard deviation) or associated estimates of uncertainty (e.g. confidence intervals)
☐	☒	For null hypothesis testing, the test statistic (e.g. *F*, *t*, *r*) with confidence intervals, effect sizes, degrees of freedom and *P* value noted<br>*Give P values as exact values whenever suitable.*
☒	☐	For Bayesian analysis, information on the choice of priors and Markov chain Monte Carlo settings
☒	☐	For hierarchical and complex designs, identification of the appropriate level for tests and full reporting of outcomes
☒	☐	Estimates of effect sizes (e.g. Cohen's *d*, Pearson's *r*), indicating how they were calculated

Our web collection on statistics for biologists contains articles on many of the points above.

Software and code

Policy information about availability of computer code

Data collection	We used the commercially available softwares Metamorph Imaging, and Leica LasX to record data from microscopes. Simulation data was generated using a modified version of Cortical Sim, which is available upon request from Dr. Eva Deinum.
Data analysis	Data was analysed using the following softwares: Fiji/ImageJ (multiple version), FibrilTool (originally published version from Boudaoud et al., Nature Protocols, 2014), Matlab (R2020b and later versions), Cortical Sim (modified version available upon request from Dr. Eva Deinum), Microsoft Excel, and the free tracking software FIESTA (1.05.0000).<br>We used the following websites to perform statistical testing and statistical data representation: PlotsOfData (https://huygens.science.uva.nl/PlotsOfData/), PlotTwist (https://huygens.science.uva.nl/PlotTwist/), and GraphPad (for t-tests; https://www.graphpad.com/quickcalcs/ttest1.cfm)

For manuscripts utilizing custom algorithms or software that are central to the research but not yet described in published literature, software must be made available to editors and reviewers. We strongly encourage code deposition in a community repository (e.g. GitHub). See the Nature Research guidelines for submitting code & software for further information.

Data

Policy information about availability of data

All manuscripts must include a data availability statement. This statement should provide the following information, where applicable:

- Accession codes, unique identifiers, or web links for publicly available datasets
- A list of figures that have associated raw data
- A description of any restrictions on data availability

All relevant imaging and computer simulation data supporting the findings of this study are available either within the article, its Supporting Information document, the source data file or upon request from the corresponding authors.

Field-specific reporting

Please select the one below that is the best fit for your research. If you are not sure, read the appropriate sections before making your selection.

- [x] Life sciences
- [ ] Behavioural & social sciences
- [ ] Ecological, evolutionary & environmental sciences

For a reference copy of the document with all sections, see nature.com/documents/nr-reporting-summary-flat.pdf

Life sciences study design

All studies must disclose on these points even when the disclosure is negative.

Sample size	There was no specific statistical method used to determine sample size. We used sample sizes that yielded sufficient statistical power in previous studies from our lab such as Endler and Kesten et al., CELL (2015), Schneider et al., Plant Cell (2017), Watanabe and Schneider et al., PNAS (2018), and Kesten and Wallmann et al., Nature Communications (2019).
Data exclusions	No samples or recordings were excluded, except when samples showed (non-linear) drift or other changes during image acquisition and this could not be corrected.
Replication	The vast majority of experiments contain at least three technical replicates with multiple biological replicates each. In cases where only one technical replicate was acquired, the number of biological replicates was significantly large to justify proper statistical comparison to the control samples. All replicates were independent from each other.
Randomization	Plants were always randomly distributed in the growth chambers and treatment areas. Kymograph analyses to measure microtubule dynamic parameters were displayed in a randomized order so that assignment to band or gap was not possible.
Blinding	Where possible, we automatized image analysis. For the measurement of plant features, investigators were not blinded since this is not relevant to this study. In this case, data was always collected according to the genotype of the plants. In simulations, always at least 100 runs were analyzed to estimate errors (16% and 84% confidence intervals) and no selection took place. This excludes the classification of microtubule arrays into transverse and co-aligned, transverse, mis-aligned, and longitudinal, where we wanted to achieve this grouping on purpose.

Reporting for specific materials, systems and methods

We require information from authors about some types of materials, experimental systems and methods used in many studies. Here, indicate whether each material, system or method listed is relevant to your study. If you are not sure if a list item applies to your research, read the appropriate section before selecting a response.

Materials & experimental systems

n/a	Involved in the study
x	Antibodies
x	Eukaryotic cell lines
x	Palaeontology and archaeology
	x Animals and other organisms
x	Human research participants
x	Clinical data
x	Dual use research of concern

Methods

n/a	Involved in the study
x	ChIP-seq
x	Flow cytometry
x	MRI-based neuroimaging

Animals and other organisms

Policy information about studies involving animals; ARRIVE guidelines recommended for reporting animal research

Laboratory animals	not applicable
Wild animals	not applicable
Field-collected samples	not applicable
Ethics oversight	not applicable

Note that full information on the approval of the study protocol must also be provided in the manuscript.
